# Acceptability and effectiveness of stationary bike intervention on health outcomes among older adults: a systematic review of intervention studies

**DOI:** 10.1186/s12877-025-06757-0

**Published:** 2026-01-13

**Authors:** Taiyeba Akter, Md. Moneruzzaman, Kellina Maduray, Manzur Kader

**Affiliations:** 1 Department of Sport Science and Sport, University of Erlangen- Nuremberg, Erlangen, Germany; 2 Department of Medical and Translational Biology, Umeå University, Umeå, Sweden; 3 School of Rehabilitation Medicine, Capital Medical University, Beijing, China; 4 China Rehabilitation Research Center, Beijing Bo’ai Hospital, Beijing, China; 5 Department of Cardiology, Qilu Hospital of Shandong University, Jinan, China; 6 Department of Medical Science, School of Health and Welfare, Dalarna University, Falun, 791 88 Sweden

**Keywords:** Stationary bike, Older adults, Physical activity, Health outcomes, Acceptability, Effectiveness, QOL, VA

## Abstract

**Background:**

Promoting physical activity (PA) among older adults is challenging due to physical limitations and varying levels of motivation. Stationary biking is a safe, non-weight-bearing form of exercise that is well-suited to this population. Although several studies have investigated stationary bike (SB) interventions, there is limited synthesis regarding their focus, effectiveness, and acceptability. This study aims to identify the primary focus areas of published SB interventions and evaluate their acceptability and impact on health outcomes in older adults.

**Methods:**

Following PRISMA guidelines, a comprehensive search of seven databases was conducted up to June 18th, 2023, without restrictions on publication year. After duplicate records were removed, two independent reviewers screened studies in a two-phase process. Eligible studies included original intervention research reporting both baseline and post-intervention outcomes, in which stationary biking was employed either as a health assessment tool or as an intervention modality among individuals aged 60 years or older.

**Results:**

Out of 8,022 studies, 47 English-language articles were included: 28 Randomized Controlled Trials (RCTs) and 19 (40.43%) Non-RCTs, including Pretest-post-test and Quasi-Experimental studies. The most common focus points of the included studies were Cognitive function, Motor and Balance, Physiological and Psychological changes, Cardiovascular, and Executive function. The most common study populations had neurological problems (15/47, 31.91% studies). Among the 47 included studies, the highest participants’ acceptance rate was 38% (18/47 studies), while the retention rate was 15% (07/47 studies), the adherence rate was 6% (3/47 studies), and the lowest dropout rate was 13% (06/47 studies). Significant health outcomes after SB intervention included Aerobic capacity (VO_2_ max), Cycling efficiency, Cognition, Executive function, Quality of life (QOL), and Mobility (Timed up and Go test), mentioned in at least 5 studies. About 25% (12/47) of all included studies used virtual aid (VA)-assisted SB intervention and among them 9 RCTs showed better improvement in cycling efficiency, cognition, and executive function compared to non-VA groups.

**Conclusion:**

A review of 47 studies found that stationary biking shows moderate acceptability (38%) and yields significant benefits in VO_2_ max, cycling efficiency, cognition, executive function, quality of life, and mobility (Timed Up and Go test) in adults aged ≥ 60. Stationary biking especially when combined with VA, shows promise as a low-risk intervention to enhance physical and cognitive health in older adults, warranting further high-quality randomized trials to optimize its prescription.

**Supplementary Information:**

The online version contains supplementary material available at 10.1186/s12877-025-06757-0.

## Introduction

 Regular physical activity (PA) provides substantial health benefits across all age groups, particularly for older individuals, by promoting prolonged years of active and independent living. The World Health Organization (WHO) projects a significant increase in the global population aged 60 years and above, rising from 12% to 22% between 2015 and 2050, with one in six individuals worldwide expected to be aged 60 years or older by 2030 [1]. Engagement in regular physical exercise and sports throughout life positively impacts cardiovascular, respiratory, metabolic, neurological, and social well-being [2]. Consequently, the WHO recommends that individuals aged 65 years and above engage in at least 150 min of moderate-intensity aerobic physical activity per week, or 75 min of vigorous-intensity aerobic physical activity per week, or an equivalent combination of both. This regimen aims to enhance cardiorespiratory and muscular fitness, bone and functional health, and reduce the risk of non-communicable diseases (NCDs), depression, and cognitive decline [3].

Various forms of endurance exercise training, such as cycle ergometer training [4], walking or jogging [5], and treadmill training [6], have been shown to improve health outcomes. Among these, stationary bike (SB) training stands out due to its ease of use, safety, and low risk of injury [7]. Their key features include adjustable resistance levels for personalized workout intensity, ergonomic seating for comfort, and stability to reduce fall risk [7–9]. SBs are non-weight-bearing, minimizing joint stress compared to jogging or high-impact activities SB exercise is particularly beneficial for its non-weight-bearing nature, which reduces joint impact and overall bodily stress compared to jogging or other high-impact activities [8]. Furthermore, SB training requires less postural control than treadmill walking, making it a suitable option for individuals with balance issues [6]. Additionally, a study by Plante et al. [10] found that VA-assisted stationary biking enhances exercise enjoyment, cognitive function, and reduces perceived exertion in older adults. However, it’s worth noting that VA-assisted biking was not the primary focus in the existing literature and warrants further exploration. Furthermore, systematic reviews specifically addressing the overall acceptability and effectiveness of SB interventions on health outcomes, particularly among older adults aged 60 and above, are currently lacking. Acceptability ensures adherence, as older adults are more likely to continue SB exercises if they are enjoyable and accessible. Factors like ease of use, comfort, and motivation influence participation and long-term benefits. Effectiveness ensures SB exercises improve fitness, strength, and balance while providing accurate performance assessments. Without high acceptability and effectiveness, SB interventions may fail to support active aging. To address this gap, this systematic review aims to comprehensively evaluate the acceptability and effectiveness of SB interventions among older adults. Specifically, the review seeks to: (a) What evidence exists regarding the acceptability of SB among older adults, and (b) What evidence exists for the effectiveness of SB intervention on health outcomes?

In this study, acceptability is defined as participant’s acceptance, dropout, retention, and adherence rates of SB interventions. Older adults are defined as individuals aged 60 years and above [11], with further categorization into younger older adults (aged 60–75 years) and older adults (aged ≥ 76 years) based on previous research [12].

## Methodology

This systematic review was conducted following the Preferred Reporting Items for Systematic Reviews and Meta-Analyses (PRISMA) guidelines [13], ensuring transparency and rigor in the review process.

### Registration

The project was registered with the Open Science Framework (OSF) (Registration https://osf.io/wn3bp), with associated project (link 10.17605/OSF.IO/WN3BP) on April 10, 2025.

### Eligibility criteria

The eligibility criteria were established through a two-stage screening process. Initially, screening was performed based on the title and abstract, guided by specific criteria delineating studies centred on bicycles, cycling, or bicycling, particularly those involving older adults or explicitly mentioning an age range of 60 years and above.

#### Inclusion criteria

For the second stage screening, after obtaining the full texts of potentially eligible studies, the following criteria were applied for inclusion.

1. All types of quantitative studies of any duration, assessing the effectiveness of an intervention (randomised or non-randomised),
2. Outcomes measured at baseline and post-intervention, with or without follow-up,
3. The studies should focus on a subgroup of the aged population aged 60 years and above,
4. Addressing stationary biking as a health assessment tool and/or part of an intervention method, and published in the English language.

#### Exclusion criteria

The exclusion criteria for this review encompassed following parameters.

1. Observational studies, including cross-sectional association or correlation studies, were excluded due to their limited ability to establish causality,
2. Qualitative studies were excluded as they typically focus on exploring subjective experiences rather than quantifiable outcomes,
3. Unpublished or under-review articles, conference abstracts, and publications in languages other than English were excluded to ensure access to peer-reviewed, scientifically rigorous research,
4. Furthermore, studies involving forms of bicycling other than stationary bicycling, such as outdoor bicycling or indoor bicycling not part of an intervention, were excluded to maintain consistency in the intervention modality under investigation,
5. Study protocols, letters, and commentaries were also excluded from the review due to their preliminary or opinion-based nature. However, relevant references from these excluded sources were screened to identify any articles meeting the inclusion criteria, ensuring a comprehensive examination of the literature.

### Information sources

Systematic searches were conducted across seven electronic databases: PubMed, Web of Science, Scopus, Cochrane Library, SportDiscus, CINAHL, and PsychInfo, without applying any filters.

### Search strategy

The search strategy was developed in collaboration with an expert library scientist specializing in information retrieval methodologies. We systematically searched for intervention studies using SB as a health assessment tool and/or intervention method among older adults. The search included publications up to June 18, 2023, with no geographical or temporal restrictions and limited to English-language articles. We used a combination of keywords: [“old people” OR “older people” OR “elderly” OR “elders” OR “aging” OR “ageing” OR “old men” OR “old women” OR “older persons” OR “older adults” OR “seniors”] AND [“bicycling” OR “cycling” OR “biking” OR “bike” OR “bicycle”]. Detailed information about the search strategy and keywords is available in Supplementary File S1.

### Selection process

All citations were imported into Covidence systematic review software (Veritas Health Innovation, Melbourne, Australia; www.covidence.org) and an Excel sheet for the screening process and narrative synthesis. Additionally, citations were imported into EndNote X9 (Clarivate Analytics, Philadelphia, PA, USA) for reference management. A two-stage screening protocol was then implemented, starting with the assessment of each citation’s title and abstract, followed by a thorough evaluation of the full-text articles for potentially relevant studies. Each screening stage was conducted independently by two review authors (TA and MK). After completion, the authors were unblinded to each other’s decisions, and any disagreements were resolved through discussions with additional review team members (MM and KM).

### Data collection process

Data from the included studies were extracted and documented through a narrative synthesis by the two independent reviewers (TA and MK). Any discrepancies between the reviewers were discussed and resolved through consensus. Extracted data included the author’s first name, year of publication, country, age range, gender distribution, and study design (RCT or Non-RCT). Further segmentation categorized the type of SB intervention, health conditions of participants, recruitment, dropout, retention, adherence rates, and primary outcomes. However, it is noteworthy that two studies [14, 15] out of 47 could not be tabulated for outcome analysis due to missing “p” values. In such instances, evidence-based theories were judiciously applied for calculation purposes, thereby ensuring methodological rigour and consistency in data analysis. Data were organised in an Excel sheet for systematic synthesis and cross-checking.

### Data items

The primary outcome data sought included adherence to the intervention, retention rates, and adverse events. Secondary outcomes included health-related parameters such as cardiovascular fitness, strength, balance, mental health, and quality of life. Data on participant characteristics (age, gender, health status), intervention specifics (type of stationary bike, duration, frequency, and intensity), and study design were collected.

### Study quality and risk of bias assessment

In assessing the quality of randomized controlled trials (RCTs) within the scope of this study, two established evaluation tools were utilized: the “PEDro” scale [16] and the Cochrane Handbook for the Risk of Bias Tool, version 2 (ROB 2.0). The “PEDro” scale evaluates studies across eleven criteria, covering aspects such as randomization, blinding, and statistical analysis. Ratings on this scale range from 0 to 10, with scores exceeding 4 indicating fair quality, scores between 6 and 8 denoting good quality, and scores surpassing 8 indicating excellent quality studies [16].

Conversely, non-randomized studies included in this systematic review underwent assessment using the Newcastle-Ottawa Scale (NOS) [17] to gauge their quality and methodological rigor. The NOS evaluates studies based on three primary domains: selection of study groups, comparability of groups, and ascertainment of outcomes. Scores are assigned within these domains to facilitate the critical appraisal of study quality and risk of bias. The quality assessment was performed independently by two authors (MM and KM) Any discrepancies were resolved through discussion with the supervising author (MK).

### Effect measures

For each outcome, the effect measures used included adherence rates (percentage of participants completing the intervention), retention rates (percentage of participants remaining in the study until the end), and adverse events (reported incidents during the intervention). Health-related parameters like cardiovascular fitness were measured using heart rate recovery and maximum heart rate (HR) achieved during exercise sessions. Executive function was assessed using standardized cognitive tests specified in each study. Effect sizes (Cohen’s d) were calculated to determine the magnitude of the intervention’s impact where data is available. Applicable, data were extracted on exercise intervention duration, session frequency, and duration to explore their relationship with observed outcomes.

### Synthesis methods

Data were synthesized narratively due to the heterogeneity in intervention types and outcomes. Studies were grouped by intervention type, health outcomes, and participant characteristics. Thematic analysis was applied to summarize findings, and results were tabulated for clarity. No meta-analysis was performed due to variability in study designs.

### Reporting bias assessment

Due to the narrative synthesis approach, no formal assessment of publication bias was conducted.

### Certainty assessment

The overall strength of evidence was assessed considering study quality, consistency of findings, and potential biases.

## Results

### Study selection

The systematic search yielded 6,639 articles after duplicate removal via Covidence software. Following title and abstract screening, assisted by the software, 126 studies were deemed eligible for full-text review. Subsequent exclusion based on duplicate entries, full-text availability, and relevance reduced this number to 99 articles for manual assessment. Upon full-text evaluation, 47 articles met the criteria for data extraction.

### Study characteristics

Table 1 outlines the characteristics of 47 included studies, encompassing a total study population of 2,500 individuals engaged in SB interventions. Among these, 28 (59.57%) studies were RCTs, while 19 (40.43%) were non-RCTs, including Pretest-posttest and Quasi-Experimental studies. A demographic breakdown reveals that 16 (34.04%) studies [10, 18–32] targeted younger older adults aged 60 to 75 years, 5 studies (10.64%) [33–37] focused on older older adults (aged ≥ 76), and 26 (55.32%) [14, 15, 37–60] included both age groups.

**Table 1:** Characteristics of 47 included studies

Characteristics	Number of studies (*n*), Percentage (%)
Study Design	
Randomized controlled trial	28 (59.57)
Non-randomized controlled trial	19 (40.43)
Age range	
Younger older adults (60–75)	16 (34.04)
Older older adults (≥ 76)	05 (10.64)
Both	26 (55.32)
Gender	
Male	02 (4.26)
Female	04 (8.51)
Both	41 (87.23)
Health status of the study population	
Neurological problems	15 (31.91)
Healthy	14 (29.79)
Existing Comorbidities (DM, HTN, cancer, and other risk factors)	07 (14.89)
Physically inactive	04 (8.51)
Cardiovascular & Respiratory problems	04 (8.51)
Musculoskeletal problems	02 (4.26)
Not specified	01 (2.13)
Types of stationary bikes used for intervention	
Ergometer	28 (59.57)
Cybercycle	12 (25.53)
Recumbent SB	05 (10.63)
Upright bicycle ergometer	01 (2.13)
Aqua cycle	01 (2.13)
Approaches to SB intervention	
Only SB intervention	27 (57.44)
SB with virtual aid/electronic device	12 (25.53)
SB with exercise/cognitive training	07 (14.89)
SB with medication	01 (2.13)

*DM* Diabetes Mellitus, *HTN* Hypertension, *SB *Stationary Bike

Various types of SB interventions were identified, with ergometers being the most common (61.70%), followed by Cybercycle (SB with virtual aid) in 12 studies (25.53%) [27, 33, 34, 39–43, 49, 52, 54, 59], Recumbent SB in 5 studies (10.63%) [23, 36, 44, 58, 61] and upright bicycle ergometers and aqua cycles (SB in water) each appearing in 2 studies (4.26%) [31, 60] separately. The majority of studies (48.93%) exclusively utilized SB interventions, while 25.53% incorporated SB with virtual aid (VA) intervention, 14.89% combined with exercise/cognitive training, and a single study combined SB intervention with medication (see details in Table 1*)*.

**Fig. 1:**
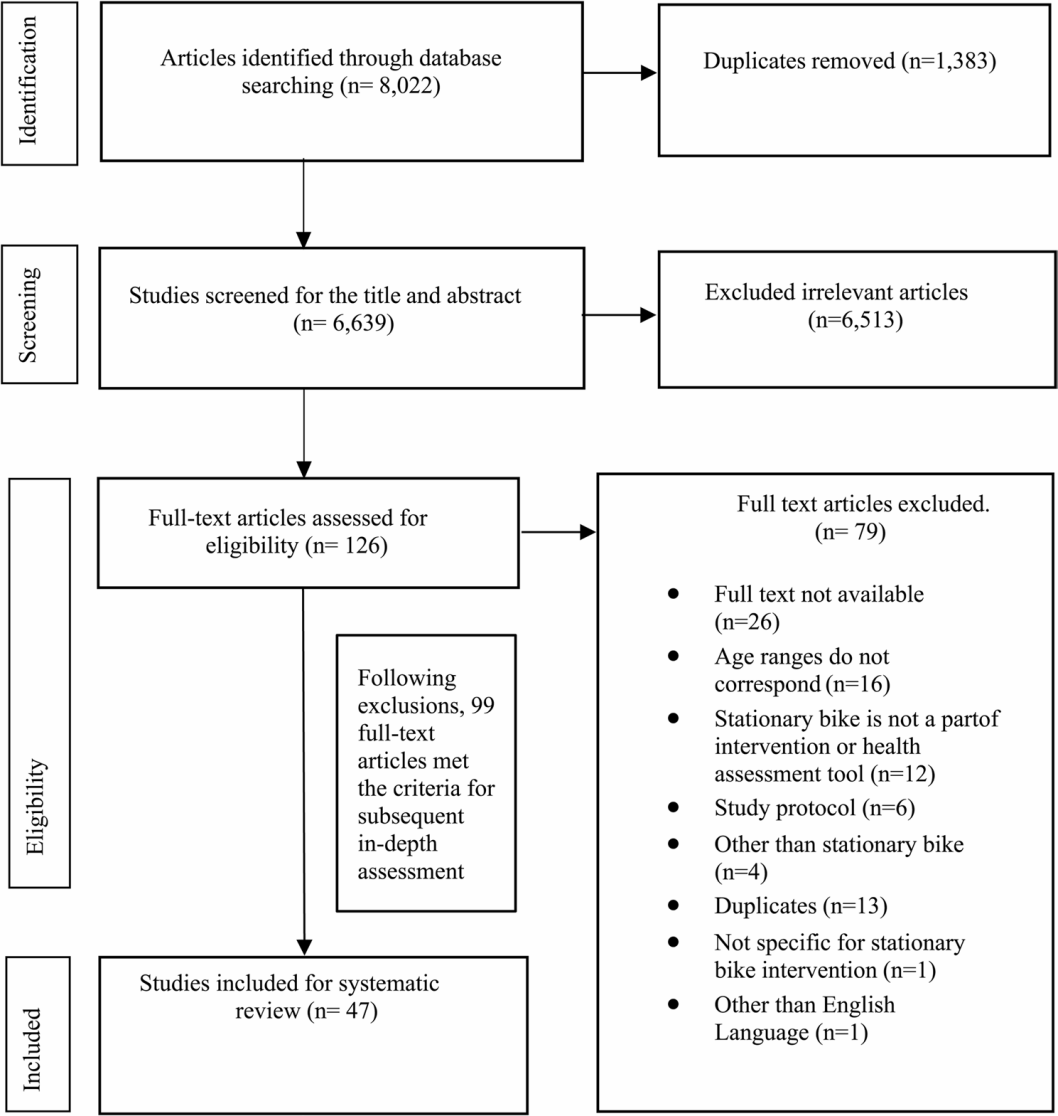
PRISMA flow diagram-systematic review: study selection process

The trend of publications, spanning from 1975 to 2023 with a half-decade interval, is illustrated in *Supplementary Fig. 1*. It encompasses combined interventions (exercise/cognitive training) and, notably, a single study combining SB intervention with medication (Fig. 1)

### Study quality and risk of bias assessment of all RCTs

We evaluated the study quality of all RCTs using the “PEDro” scale [16] and Cochrane Handbook for risk of bias tool “ROB 2.0” [16]. The “PEDro” scale evaluates studies on eleven variables and assigns scores between 0 and 10; a score above 4 indicates fair, while 6 to 8 indicates a study of good quality, and above 8 indicates an excellent quality study.

Regarding the PEDro scale, sixteen studies [18, 22, 23, 28, 30–32, 34–39, 44, 50] scored between 6 and 8, and nine studies [26, 27, 29, 32, 41–43, 57, 60] scored between 4 and 5. Please refer to Supplementary Table 2 for further details.

Regarding RoB 2.0, eleven studies [18, 22, 23, 28, 30–32, 39, 53, 54, 58] are considered as low risk of bias. Two studies [35, 42] had a high risk of bias due to the randomization process and missing outcome data. Other studies [26, 27, 29, 33, 34, 36–38, 41, 43, 44, 50, 56, 57, 60] had overall some concerns because of randomization, selection of reported results, and measurement of the outcome. *(see Supplementary Table 3* for further details).

### Study quality of all Non-RCTs

Among the remaining 19 non-RCT studies assessed with the Newcastle-Ottawa Scale, nine studies [19, 24, 25, 37, 47–49, 51, 59] achieved the highest scores of 9, indicating strong performance across assessment criteria and robust methodology. Following closely were other studies [9, 20, 21, 52] with a score of 8, highlighting commendable methodological quality. Four studies [14, 40, 46, 55] received a score of 7, indicating a good level of quality in design and execution. One study [45] scored 5, reflecting a lower level of methodological quality, while another study [15] scored 3 due to design limitations and lack of methodological details *(see Supplementary Table-4 for further details).*

### Results of individual studies

**Fig. 2:**
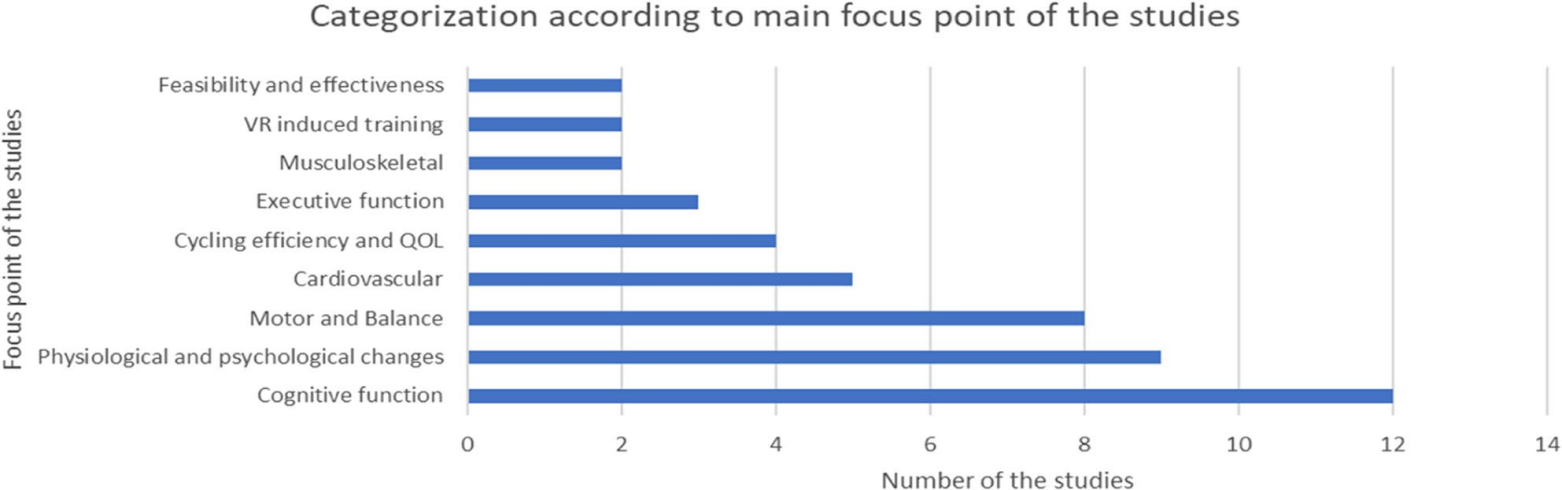
Categorization according to focus points of the studies. *QOL *Quality of Life, *VR *Virtual reality

Figure 2 illustrates the primary areas of investigation in the 47 included studies. Cognitive function emerged as the most frequent focus, with 12 studies [24, 27, 33, 34, 39, 40, 42, 43, 47, 54, 59, 61]. Other prominent categories include Motor and Balance (seven studies) [10, 22, 32, 38, 44, 48, 51], Physiological and psychological changes (nine studies) [15, 18, 20, 30, 45, 50, 53, 56, 58], Cardiovascular (five studies) [19, 25, 28, 29, 60], Executive function (three studies) [23, 26, 36], Cycling efficiency and Quality of life (four studies each) [14, 21, 46, 52], VA-induced training, and Feasibility and effectiveness (four studies each) [15, 35, 41, 49]. Additionally, studies related to the Musculoskeletal system were represented by two studies [31, 55].

**Fig. 3:**
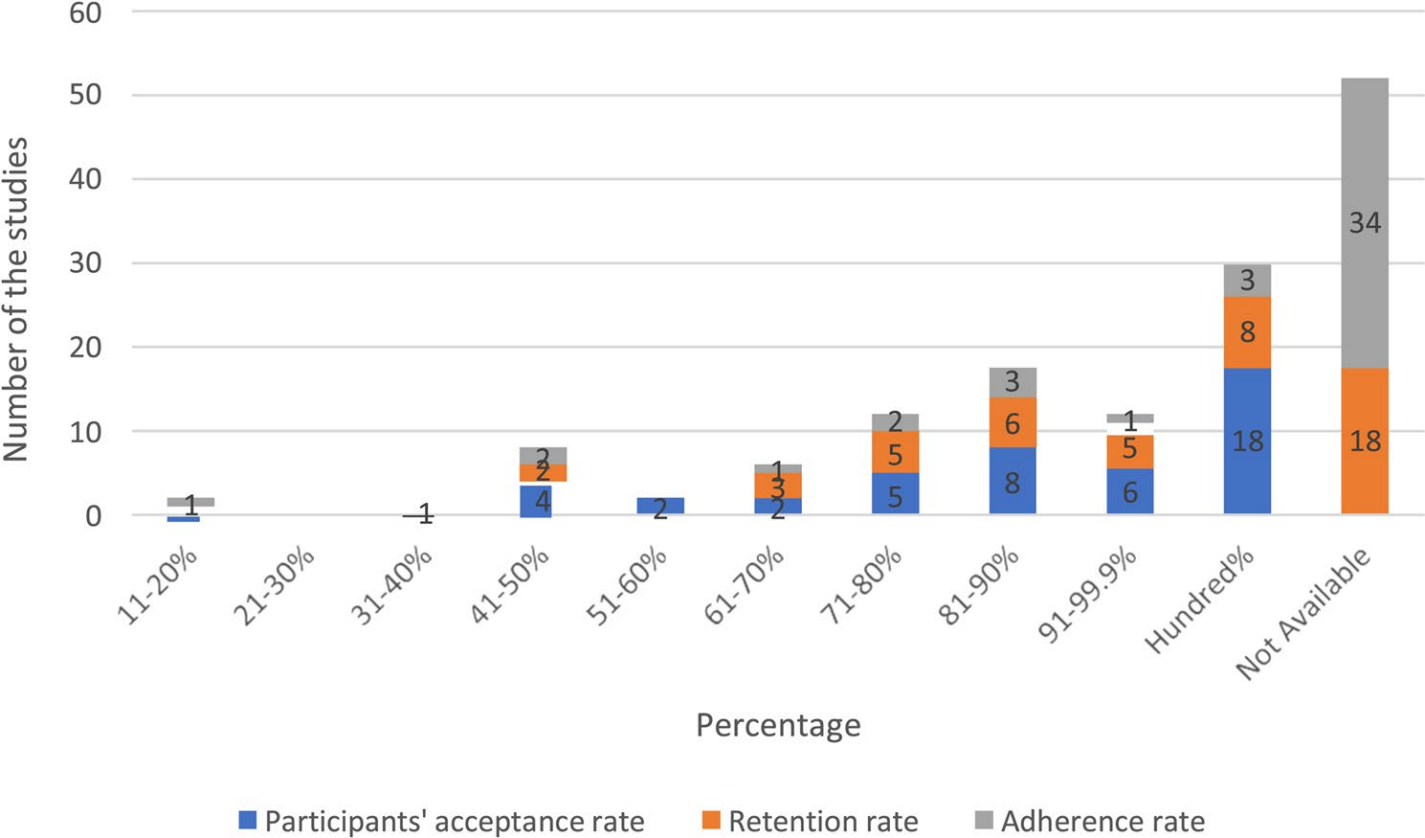
Acceptance, retention, and adherence rate of stationary bikes among the study population

**Fig. 4:**
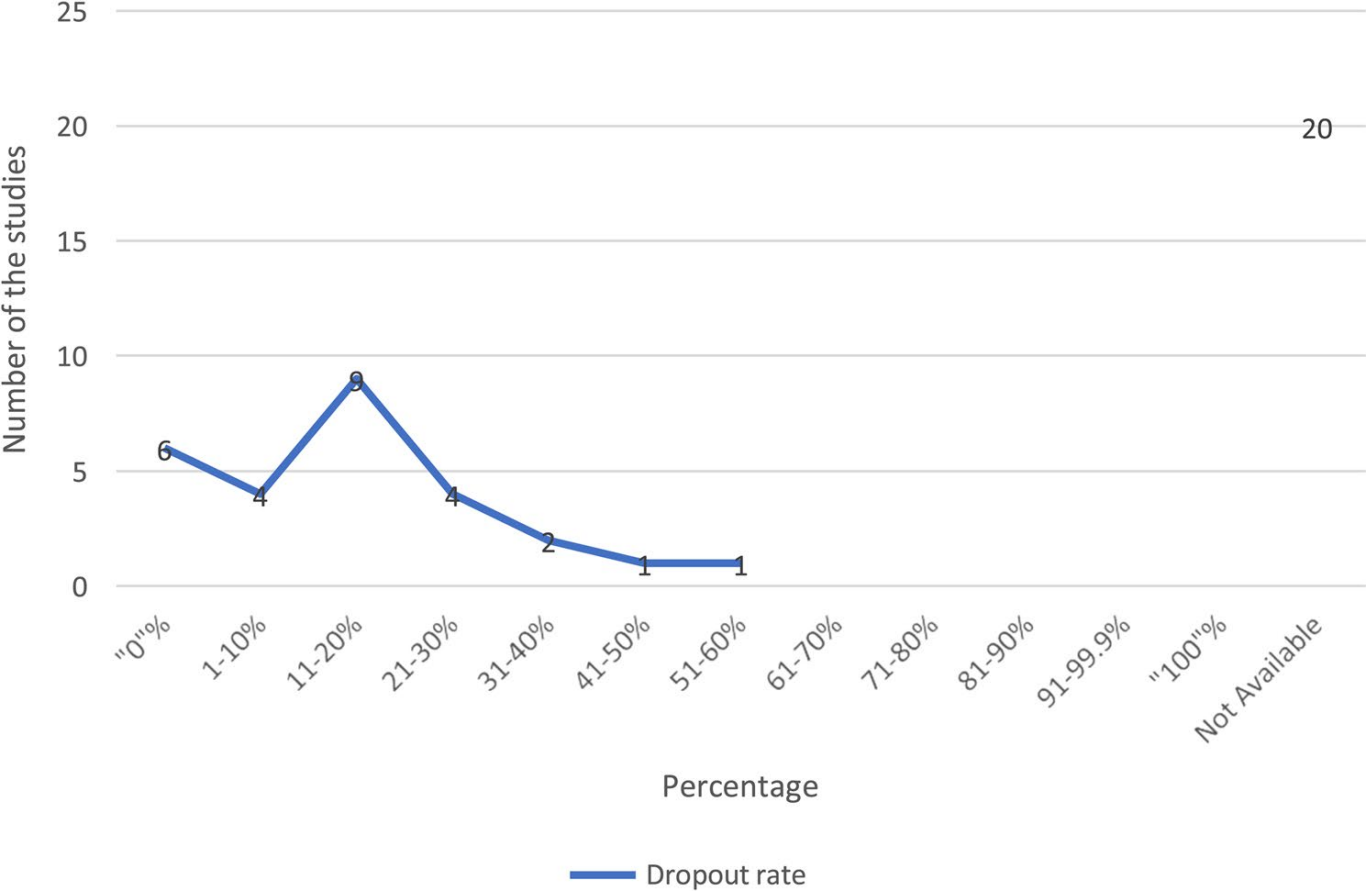
Dropout rate of stationary bikes among study population

Evidence related to the acceptability of SB intervention: Among the 47 included studies, 18 studies [18, 20, 21, 24, 25, 27, 29, 30, 36, 41, 42, 44, 46, 50–52, 56] reported a 100% acceptance rate, 7 studies [27, 32, 43, 49, 53, 54, 57] had a 100% retention rate, and 3 studies [32, 39, 54] reported full adherence. Additionally, 6 studies [32, 43, 49, 53, 54, 57] recorded a 0% dropout rate (see Table 2; Figs. 3 and 4)*.* The second highest acceptability range (81–90%) was observed in 17 studies, with acceptance reported in 8 [19, 23, 38, 40, 47–49, 60], retention in 6 [19, 21, 23, 34–36], and adherence in 3 [15, 23, 49]. In the 91–99.9.9% range, 12 studies showed acceptance in 6 [22, 32–34, 36, 61], retention in 5 [20, 28, 31, 56, 60], and adherence in 1 [37]. The 71–80% range was observed in 12 studies, with acceptance in 5 [37, 39, 43, 55, 59], retention in 5 [15, 22, 39, 40, 55], and adherence in 2 [39, 56] (see Table 2).

Lower adherence rates were found in 8 studies (41–50%) with acceptance in 4 [10, 26, 28, 35], retention in 2 [36, 42], and adherence in 2 [42, 45]. The lowest range (61–70%) was found in 6 studies, with acceptance in 2 [31, 58], retention in 3 [33, 37, 48], and adherence in 1 [55] (see Table 2)*.*

Dropout rates ranged from 0% to 60%, with 9 studies [19, 21–23, 34, 35, 47, 55, 61] reporting an acceptable 11-20% dropout rate (see Fig. 4)*.* A total of 19 studies [19–23, 28, 31, 32, 34, 35, 43, 47, 49, 53–55, 57, 60, 61] had dropout rates below 20%, while 8 studies [15, 33, 36, 37, 39, 40, 42] exceeded 20% (see Table-2). Three studies [23, 32, 49] demonstrated feasibility with ≥80% acceptance, retention, and adherence, and <20% dropout.

Overall, acceptability rates were 38% for acceptance (18/47 studies), 15% for retention (7/47), 6% for adherence (3/47), and 13% for dropout (6/47).

### The effectiveness of SB interventions across health outcomes

The effectiveness of SB interventions varied across health outcomes. Table 2*(see also supplementary Table-1)* represents an overview of findings from all 47 included studies. As most studies implemented SB in combination with VA, outcomes are reported by intervention modality (SB + VA vs. SB alone) to enable clearer interpretation of their relative effects.

**Table 2:** Extracted data from 47 included studies

Included Articles	Acceptability of stationary bike intervention							Description of effectiveness				
-First Author (Year of publication) - (Study design)	Offer made to the eligible Participants (n)	Number of people randomised. (n) *(for RCT/Quasi ex.)* Or Number of eligible participants accepted the offer (n) *(other than RCT/Quasi ex.)*	Participant’s acceptance rate: *RCT/Quasi ex.: [Number of people randomised (n)/offer made to the eligible (n)] x 100 (%)* *Other than RCT/Quasi ex.: [Offer accepted by the eligible (n)* */Offer made to the eligible (n)] x 100 (%)* *(Arring et al.*,* 2022*	Participants left the intervention (n)	Drop-out rate: *For RCT/Quasi ex.: [Participants Left the intervention (n)* */no. of participants started stationary bike intervention (n)] x 100 (%)* *Other than RCT/Quasi ex.: [Participants left the intervention (n)/Offer accepted by the eligible (n)]x* *100(%)* *(Gupta et al.*,* 2016)*	Retention rate: *RCT/Quasi ex.: No. of completers(n)/p articipants started stationary bike intervention (n)] x100(%)* *Other than RCT/Quasi ex.: [No. of completers (n)/offer accepted by the eligible (n)] x 100 (%)* *(Yu et al.*,* 2013)*	Adherence rate to the prescribed stationary bike exercise (%)	Health status of the study population during enrolment process	Disease/condition for the intervention	What found? or ↑/↓/no improvement after stationary bike intervention	Types of Stationary bikes with added features for intervention purposes	
Abbas (2022)[38] (RCT)	68	60 *(Started SB intervention: 20*,* Completers N/A)*	88%	N/A	N/A	N/A	N/A	Moderate to severe dementia	Balance and mobility	Significant increase of balance and reduction of mobility among ergometer, and “ergometer & HIFE group” when compared with “pre- treatment” group.	Virtual aid assisted cycle ergometer	
Anderson- Hanley, C. (2012)[40] (RCT)	102	79 *(Started SB intervention:38*, *Copmleters:30)*	77%	8 (*Non-compliant to bike:3*, *others:5)*	21%	79%	80%	Healthy	Executive function	Better executive function among SB group than traditional SB exercisers.	Recumbent stationary bike with virtual aid	
Anderson- Hanley (2011)[39] (Non-RCT), Quasi experimental)	23	20 *[Started SB intervention:13*,* Completers:10 (T2DM)]*	87%	3 *(Moved out: 1*,* medical complications:2* *)*	23%	77%	100% *(All followed the 3 months of monitored* *exercise))*	Type-2 Diabetes Mellitus	Executive function	T2DM patient exhibited significant gain on Colour trails test to detect executive function than non-DM patient.	Cyber cycle vs. ergometer	
Anderson- Hanley (2018)[42] (RCT)	111	111 *(Started SB intervention:46*,* Completers:23)*	100%	23 *(Knee pain*,* motion sickness*,* Illness/injury: 15*,* too difficult* *to perform: 4*,* medical complications:4* *)*	50%	50%	50%	Impaired cognition (Mild to moderate)	Executive function	Exer-tour group (exercise bike with virtual tour) yielded significant improvement on executive function compared to *exer- score* (pedal-based videogame) group.	Recumbent stationary bike with virtual aid	
Anderson- Hanley (2012)[41] (RCT)	14	14	100%	N/A	N/A	N/A	N/A	Healthy	Virtual reality induced competitiveness on exercise effort	Virtual social facilitation increased exercise effort among competitive exercisers.	Cyber-cycle	
Antunes (2015)[18] (RCT)	46	46 *(Started SB intervention: 23)*	100%	N/A	N/A	N/A	N/A	Healthy	Memory and Physiological function	Significant improvement in memory, decreased blood viscosity, and higher aerobic capacity in ergometer group compared to control (no intervention) group.	Ergometer (Stationary Bike)	
Barcelos (2015)[43] (RCT)	64	48	75%	0	0%	100%	42%	Healthy	Executive function	Exergaming group performed better interactive mental challenge than others.	Stationary bicycle with virtual aid	
Bellumori (2017)[44] (RCT)	26	26 *(Started SB intervention:14)*	100%	N/A	N/A	N/A	N/A	Healthy	Mobility	Relatively low dose speed based stationary bike group improved neuromuscular function and tests of mobility compared to control (daily activities) group.	Recumbent stationary bicycle	
Brisswalter (2014)[46] (Non-RCT), Non-randomised comparative study	20	20 *(≥ 60 yrs.)*	100%	N/A	N/A	N/A	N/A	Healthy Triathlete	Cycling efficiency	Cycling efficiency continued to decrease beyond 60 (60–69) years when compared with 50– 59 years group.	Ergometer	
Buccola [45](1975) (Non-RCT), Non-randomised comparative study	36	20	56%	N/A	N/A	N/A	N/A	Not specified	Physiological and personality changes	Increased VO2max, reduced BP and weight, and cyclers did not change any personality factors over walk-joggers group.	Ergometer	
Callow (2022)[47] (Non-RCT), Pretest-posttest)	35	30	86%	5 *(Poor performance:4*,* Failed to* *respond: 1)*	17%	N/A	N/A	Healthy	Memory	Better mnemonic discrimination performance following ergo cycle exercise group.	Cycle ergometer	
Cicek (2020)[48](Non-RCT)	67	58 *(Started SB intervention:21*,* Completers: 14*)	87%	7 *(Discontinued* :*6 others:1)*	33%	67%	N/A	Existing comorbiditi es	Mobility and balance	Ergometer & treadmill-based program found less effective than video-based program on mobility but found effective on balance test.	Ergometer and treadmill	
Colombo (2023)[49] (Non-RCT), Pretest-posttest	14	12	86%	0	0%	100%	86%	Chronic Obstructive Pulmonary Disease (COPD)	Acceptability and user experience of VA based endurance training program.	Exercise capacity (walking test) significantly improved after VA based exercise intervention.	Cycle ergometer with virtual Aid	
Cunha (2021) [19] (Non-RCT), Non-randomised comparative study	84	71 *(Started SB intervention: 17*, *Completers 15)*	85%	02 *(Due to time commitment)*	12%	88%	N/A	Hypertensiv e with a stable BP for last 3 months	Effect of BP after exercise intervention	After 30 mints of exercise only water- based exercise (swimming) lowered SBP when compared with ergo-cycle group.	Cycle ergometer	
D’Cunha (2021)[33] (RCT), Randomised crossover	11	10	91%	3 *(Due to lower* *body discomfort)*	30%	70%	N/A	Impaired cognition (Mild to moderate)	Cycling efficiency	Lower response in the SB intervention than the control condition	Stationary bike with virtual aid	
Emery (1994)[20] (Non-RCT)	64	64	100%	3 (not mentioned clearly)	5%	95%	N/A	Chronic obstructive pulmonary disease (COPD)	Physiological and psychological	Improved psychological and physiological function.	Ergometer (Stationary bike)	
Ferrai (2004) [21] (Non-RCT), Pretest-posttest	32	32	100%	4 *(Refused to continue-2)*	13%	88%	N/A	COPD	Cycling efficiency and QOL	Exercise tolerance and QOL improved (No variation observed in pulmonary function test).	Ergometer with a mechanical brake	
Ferraz (2018)[22] (RCT)	76	72 *(Started SB intervention:25*)	95%	5 *(Non- adherent:2*,* medical complications:3* *)*	20%	80%	N/A	Diagnosed PD	Walking capacity among PD patient	Improved walking capacity among ergometer, functional training and exer-gaming group.	Ergometer (Stationary bike)	
Gaesser (2018)[14] (Non-RCT), Non-randomised comparative study	82 *(≥ 60 yrs.*)	82	100%	N/A	N/A	N/A	N/A	Healthy	Cycling efficiency	Cycling efficiency was not different when compared with younger group.	Ergometer	
Gitlin (1992)[50] (RCT)	267	267 *(Both intervention and control group* *receive ergometer* *intervention but in different duration*,* total completed* *intervention: 254)*	100%	13 *(Lack of interest-9*,* and* *medical complication-4)*	N/A	N/A	N/A	Healthy	Physiological and psychological	Better QOL found after ergometer exercise intervention.	Ergometer	
Hill (2015)[51] (Non-RCT), Non-randomised comparative study	09	09	100%	N/A	N/A	N/A	N/A	Physical inactivity	Walking on postural sway	Post ergometer exercise balance impairments lasted for 10 min.	Ergometer	
Hou (2023)[23] (RCT)	154	128 *(Started SB intervention 44*,* Completers:38)*	83%	06 (Health problems:3, Other reasons:3)	14%	86%	86%	Hypertensive	Memory	significant improvements in working memory when compared with control group (without exercise)	Recumbent stationary bike	
Joyce (2014)[24] (Non-RCT), Non-randomised comparative study	12 *(≥ 61 yrs.*)	12	100%	N/A	N/A	N/A	N/A	Healthy	Cognition	Older adults adopted more cautious. strategies compared to younger adults (23 ± 2 yrs.)	Ergometer	
Katyal (2003)[25] (Non-RCT), Non-randomised comparative study	24 *(≥ 60 yrs.*)	24	100%	N/A	N/A	N/A	N/A	Physical inactivity	Circulatory function among post- menopausal women	Age and hormone replacement did not affect circulatory function after bike intervention.	Ergometer	
Kwan (2021)[34] (RCT)	18	17 *(Started SB intervention: 9*, Completers: 8)	94%	01 (repetitive VA sickness)	11%	89%	n/a	Impaired cognition	Cognitive function	VA stimulating motor-cognitive training effectively enhances the cognitive function compared to control (non-VA motor training) group.	Cycle ergometer	
Lebeau (2020)[26] (RCT)	71	35	49%	n/a	n/a	n/a	n/a	Healthy	Executive function	Participants performed better on the Trail Making Test and Stroop test after the fitness test compared to their baseline.	Cycle ergometer	
Lee (2014)[10] (Non-RCT), Non-randomised comparative study	24	12 *(SB intervention offered for 12* *only)*	50%	N/A	N/A	N/A	N/A	Healthy	Effect of gait and balance	Stationary bike group improved balance than treadmill group.	Ergometer (Stationary bike)	
Loggia (2021)[52] (Non-RCT), Non-randomised comparative study	12	12	100%	N/A	N/A	N/A	N/A	Impaired cognition (Mild to moderate)	Virtual reality upon cycling tendency	Most participants would rather repeat cycling sessions with VA than without VA.	Ergometer with or without VA	
Lopez-Garcia (2019)[27] (RCT)	49	49	100%	N/A	N/A	100%	N/A *(High adherence but did not mention* *percentage)*	Healthy	Cognition	Ergometer with video game intervention acutely improved choice reaction time.	Ergometer with or without video game	
Madden [28](2009) (RCT)	36	18 (Completers:17)	50%	1 (Other reasons)	6%	94%	N/A	Existing comorbidity	Arterial stiffness among non-communic able diseases (NCDs) patients	A relatively short aerobic exercise intervention in older adults can reduce arterial stiffness among NCDs patient.	Ergometer with treadmill	
Mahajan (2021)[53] (RCT)	34	13 *(Started SB intervention: 6)*	38%	0	0%	100%	N/A	Sleep impaired	To check the feasibility of the intervention	Ergo cycle training is feasible for older adults to support a full scale RCT for sleep intervention evident by > 80% as retention rate.	Ergometer	
Miki (2014)[54] (RCT)	146	78 *(Started SB intervention: 38)*	53%	0	0%	100%	100% (*All participants completed all the sessions with fun)*	Breast or prostate cancer	Speed feedback therapy on Cognition	Speed-feedback therapy with ergometer group had higher mean FAB score than the control group and suggested an effective intervention for cognition.	Ergometer with visual speed back therapy	
Morita (2013)[29] (RCT)	131	131	100%	N/A	N/A	N/A	N/A	Existing comorbidity	Cardiovascula r effect on BP, and BMI.	Decreased BP and BMI but more in women than man.	Ergometer	
Nocera (2020) [30] (RCT)	37	37 *(Started SB intervention*: *25)*	100%	N/A	N/A	N/A	N/A	Physical inactivity	Physiological and psychological	Aerobic exercise group improved cardiac fitness and mobility after intervention.	Ergometer	
Pauwels (2018)[55] (Non-RCT), Pretest-posttest	20	15	75%	3 (*Balance problem:2*,* ergo-cycle was* *bigger:1)*	20%	80%	67%	Lumber stenosed participants	Home based cycling on lumber stenosis	Self-reported clinical improvement, with reduced radicular pain and disability.	Ergometer	
Posner (1992) [56] (RCT)	247	247 *(Started SB intervention: 166*)	100%	N/A	N/A	93%	75% *(Completed prescribed exercise)*	Physical inactivity	Physiological response on endurance training	Improved physiological function (VO2max) among exercise group and decreased in control group.	Ergometer	
Rezasoltani (2020)[31] (RCT)	47	32 *[Started SB intervention (aqua bike)*: *15]*	68%	1 (Declined to continue: 1)	7%	93%	N/A	Knee osteoarthriti s	Effect on Pain for knee OA participants after aqua cycling	Aqua-cycling group experienced significant pain reduction (*p* < 0.001) compared to control (pain relieving medicine) group.	Aqua cycle	
Ridgel (2019)[57] (RCT)	58	16 *(Started stationary bike intervention:08*,* Completers:08)*	18%	0	0%	100%	N/A	Diagnosed Parkinson’s disease	High cadence on motor function of PD patient.	Improved mobility and motor function after high cadence ergo cycling compared to control (stretching) group.	Virtual aid assisted ergometer (stationary bike)	
Salisbury (2022)[58] (RCT)	67	47	70%	N/A	N/A	N/A	N/A	Diagnosed Alzheimer disease with dementia	Physiological Changes	significantly increased peak oxygen (VO2Peak) by 1.28 compared to the control (stretching) group.	Recumbent Stationary bike	
Schwarck (2021)[59] (Non-RCT), Pretest-posttest	19	14	74%	N/A	N/A	N/A	N/A	Diagnosed Alzheimer disease patients with dementia	Cognition	Reaction time in the picture recognition task significantly decreased.	Ergometer	
Tollár (2019)[32] (RCT)	88	83 *(Started SB intervention: 27)*	97%	0	0%	100%	100%	Participants with mobility difficulties	Effects of exercise on motor and balance, and QOL	Activities of daily living, berg balance Score and gait index improved more in exergaming group than ergometer group.	Ergometer (Stationary bike)	
VanRoie (2017)[35] (RCT)	198	95 *(Started SB intervention of strict coach together with minimal contact coach: 71*, *Completers: 61)*	48%	10 *(Medical complications: 6*,* Loss of* *interest: 2*, *others: 2)*	13% *(Directly stated)*	86%	20%	Healthy	Feasibility and long-term effectiveness of Ergo- cycling	Ergometer intervention ↑QOL among intervention group irrespective of types of supervision (Strict, minimal, and no supervision/controlled cognition)	Ergometer	
Willenheimer (1998)[60] (RCT)	61	50 *(Started SB intervention: 23)*	82%	1 *(Developed rheumatoid arthritis)*	4%	96%	N/A	Diagnosed Alzheimer disease	Effect of exercise training on heart failure	Supervised exercise training was safe and beneficial in heart failure patients specially men aged ≤ 75years with ischemic aetiology.	Upright bi- cycle ergometer	
Wu (2023)[36] (RCT)	52	52 *(Started SB intervention: 26*, *Completers: 11)*	100%	15 (Refused to exercise11, fall:1, medical complications:3 ).	58%	42%	N/A	Moderate dementia	Cognitive Function	Significantly shorter reaction time in exergame post intervention compared to recumbent bike exercise group.	Recumbent bike	
Yu (2011) [37] (Non-RCT), Pretest-post test	11	8	73%	3 (Exercise related anxiety- 1)	37.5%	62.5%	97.9% *(Average taken)*	AD	Cycling intervention on physical fitness	Significant reduction of heart rate.	Ergometer	
Yu (2021)[61] (RCT)	103	96 *(Started SB intervention:64*,* Completers: 53)*	93%	11 (*Withdrew:5*,* medical complications:2* ,*other reasons:2))*	17%	83%	N/A	AD	Cognitive effect on AD patient	Aerobic exercise failed to show any superior cognitive effect over stretching.	Ergometer	
Yu (2013)[15] (Non-RCT), Pretest-posttest	29	28 *(Completers: 22)*	97%	06 *(Other reasons)*	21%	79%	86.4%	AD	Recruitment, retention, and adherence to 6-month cycling among AD.	Recruitment relies on community partnership and exercise safety improves retention and adherence.	Ergometer (Stationary bicycle)	

*N* = 47 studies; *RCT* Randomized controlled trial, *Non-RCT* Non randomized controlled trial, *HIFE* High intensity functional exercise, *VA* Virtual aid, *FAB* Frontal Assessment Battery, *SBP* Systolic blood pressure, *DBP* Diastolic blood pressure, *BP* Blood pressure, *BMI* Body mass index, *N/A* Not available, *SB* Stationary bike, T2DM

### Effects on aerobic capacity (VO_2_ max)

The most commonly assessed outcome, VO₂ max, was examined in 10 studies. Of these, 4 showed significant (*p* ≤ 0.05) [18, 25, 45, 58] and 4 showed highly significant improvements (*p* ≤ 0.001) [20, 21, 30, 56] after SB intervention. Among RCTs with comparable groups, improvements were reported in 4 studies ([18, 58]: *p* ≤ 0.05; [30, 56]: *p* ≤ 0.001), while 2 RCTs found no significant changes [28, 60] *(see supplementary Table-1).* None of these RCT studies used combined (SB + VA) SB intervention.

### Effects on cycling efficiency

The second most outcome, cycling efficiency was assessed in 8 studies *(see supplementary Table-1 and Table-2)*. Among them five studies investigated cycling efficiency in older adults (≥ 60 years) by comparing VA enhanced stationary cycling with traditional non-VA cycling [27, 33, 38, 41, 57]. All five studies demonstrated significantly greater improvements in the VA groups (*p* = 0.002–0.049), with inferred effect sizes ranging from small-moderate to moderate-large (*see Appendix 1*). Notably, one study reported gains in both groups, though the effect was larger in the VA condition [57]. Additionally, one study implemented VA-assisted SB without any non-VA comparator and reported statistically significant improvements in cycling efficiency through repeated participation (*p* < 0.05) [52]. In contrast, two studies also conducted SB interventions without VA; one reported increased cycling efficiency (*p* < 0.05) [53], whereas the other observed a significant decline (*p* < 0.05) [46] *(see Suppl. Table-1).*

### Effects on cognition

Cognition was assessed in seven studies, including five RCTs [27, 34, 36, 54, 61]. Most RCTs reported small to large significant improvements (*p* ≤ 0.05 to *p* ≤ 0.001) in cognitive performance, particularly in choice reaction (CR) time, compared with control groups. Notably, two of these RCTs incorporated combined SB and VA interventions [27, 54]. Among the five RCTs, two reported decreased CR time [2, 24], while one found no cognitive benefit over stretching in Alzheimer’s disease patients [61]. Additionally, two non-RCT studies also reported improved cognition (*p* < 0.001) [24], although one did not report a p-value [2] (*see Suppl. Table-1*).

### Effects on executive function

Executive function was assessed in six studies, including five RCTs [26, 40, 42, 43, 61]. Three of these employed combined interventions (SB + VA) and reported small to moderate improvements compared with non-VA groups (*p* < 0.05 to *p* < 0.001) [40, 42, 43]. Improvements on the “Colour Trails” test were observed in three studies (*p* = 0.002, d = 0.5; 0.007; 0.02) [39, 40, 43], while the “Stroop A/C” test showed significant to highly significant gains (*p* < 0.05 to *p* < 0.001) in two studies [26, 42]. In contrast, one study reported no group difference [43], and another found no effect of ergometer training on executive function in Alzheimer’s disease patients [61] (*see Suppl. Table-1*).

### Effects on quality of life (QOL)

Five studies assessed QOL among them four studies are RCT and none of them used combined SB intervention [32, 35, 50, 60]. Two COPD studies reported highly significant improvements (*p* ≤ 0.001) following ergometer intervention [35, 50], and another study found a significant QOL improvement (7/9 score) in COPD patients post-ergometer intervention [21]. Alzheimer’s disease participants also showed statistically significant QOL gains (*p* < 0.05) [60], while one study found greater improvements with SB interventions compared to non-stationary cycling [32] *(see Suppl. Table-1).*

### Effects on Mobility, gait and balance

The Timed Up and Go (TUG) test was assessed in 5 studies. Three of them are RCT and reported significant (*p* < 0.05) mobility improvements in healthy, physically inactive, and Parkinson’s disease (PD) participants compared to control group [30, 44, 57], while one of them used combined SB intervention [57]. However, two studies found no mobility benefits [38, 48]; among them one study found no improvement when compared with non-VA SB intervention group [38]. Walking capacity improved significantly in COPD and PD patients [22, 49] but not in individuals with mobility difficulties when compared with control group [32].

Gait outcomes improved significantly in one study (*p* < 0.01) when compared with control group with combined SB intention [57], while no improvement was seen in another study on physically inactive participants [51]. The Berg Balance Scale (BBS) showed improved balance (*p* < 0.05) in 3 studies [10, 38, 48] and among them one study found better improvement when compared with non-VA SB group [38]. However, one study found no improvements among mobility-impaired individuals when compared with control group [32] *(see Suppl. Table-1).*

### Effects on memory

Memory improved significantly (*p* < 0.05; *p* = 0.012, η² = 0.07) in both healthy [18, 47], and hypertensive participants (compared with control group) [23], but no improvement observe with those with AD when compared with control group [61] (see Table 2 and see Suppl. Table-1).

### Miscellaneous findings

Blood pressure decreased significantly (*p* < 0.05) in two studies [29, 45], however, another study found unchanged [19]. Significant (*p* < 0.05) reduction of back pain mentioned in two studies [31, 55] and lowering of heart rate in another study [37]. On contrast, another study failed to show any improvement when compared with control group among AD participants [60] *(see Suppl. Table-1).*

Outcomes of RCT studies: Out of 28 RCT studies *(see Suppl. Table-1)*, positive outcomes were noted, notably improved aerobic capacity (VO_2_ max) in 4 studies (p = < 0.05) [6, 7, 18, 31]. The “Colour Trails” test improved in 2 studies (p = < 0.05) [39, 43], and the “Stroop a/c” test in another 2 studies (p = < 0.05) found improved as part of executive function respectively [10, 42]. Mobility (TUG test) showed improvement in 3 studies (p = < 0.05) [6, 44, 58]. QOL also significantly improved in 3 different studies (p = < 0.05) respectively [3, 62, 63]. Improved overall cognitive function was found in 2 different studies (p = < 0.05) without specifying the outcome of Choice Reaction (CR) time [26, 54]. Memory improved in 2 different studies (p = < 0.05) [18, 24]. Cycling efficiency with and without virtual aid (VA) increased in 2 different studies distinctly (p = < 0.05) [16, 41]. However, Balance (BBS test), Walking capacity, Gait, Pain reduction, & Lowering of BP showed the least significant improvement, each presented in a single study separately ((p = < 0.05) [14, 30, 38, 57, 58].

Upon analysing all the RCTs, several outcomes failed to show any improvements. Notably, Cognitive function was stated in 3 studies, among them 2 showed increased Choice Reaction time indicating decreased cognitive function (p = < 0.05) [11, 64], and another study showed an overall decrease in Cognitive function (p = < 0.05) [61]. The Stroop test and overall executive function as part of Executive function, and Aerobic Capacity both failed to show any improvement in 2 different studies individually (*p* < 0.05) [28, 43, 60, 61]. Additionally, Cycling with VA and Mobility (TUG) failed to show any improvement as stated in 2 different studies (p = < 0.05) [33, 38]. However, QOL, Balance, and Walking Capacity each of the outcomes failed to show any improvement as stated in a single study (p = < 0.05) [65].

### VA-enhanced SB interventions in older adults among RCTs

Table 3 summarizes RCTs in adults aged ≥ 60 years, demonstrating that SB with VA interventions consistently yielded superior outcomes compared to non-VA conditions. VA protocols improved walking distance and perceived exertion (*p* < 0.05, small-moderate) [27], engagement and exertion in dementia (*p* = 0.012, small; *p* < 0.05, small-moderate) [33, 38], executive function in diabetes (*p* < 0.05, small-moderate) [40], at-risk/mild cognitive impairment (MCI) cohort (*p* = 0.049, small-moderate) [42], competitiveness in community-dwelling adults (*p* = 0.003, moderate) [41], and engagement in healthy older adults (*p* < 0.05, small-moderate) [43]. Additional benefits included adherence and cognitive function in cancer (*p* < 0.05, small-moderate) [54] and enhanced cycling efficiency in Parkinson’s disease (VA: *p* = 0.002, moderate; non-VA: *p* = 0.012, small) [57]. Overall, effects consistently favoured VA interventions.

**Table 3:** Effectiveness of SB with VA vs Non-VA intervention outcome among older adults (≥60 years) in RCTs

First Author (Year)	Sample (≥ 60 yrs)	VA	Non-VA	Reported p-value	Inferred Effect Size Category*	Direction of Effect
López-García (2019) [27]	Healthy older adults	Longer distance, higher perceived effort, and ↑cognition	Shorter distance & lower perceived effort	p < 0.05	Small-moderate	Favours VA
D’Cunha (2021) [33]	Older adults with dementia	↑ Perceived exertion, better engagement	No improvement	p = 0.012	Small	Favours VA
Abbas (2022) [38]	Dementia cohort	Significant ↑ in cycling efficiency	No improvement	p < 0.05	Small-moderate	Favours VA
Anderson-Hanley (2012)[40]	Older adults with diabetes	↑ Effort and executive function	No improvement	p < 0.05	Small-moderate	Favours VA
Anderson-Hanley (2011)[41]	Community-dwelling older adults	↑ Exercise effort & competitiveness	Minimal change (p value not reported)	p = 0.003	Moderate	Favours VA
Anderson-Hanley (2018)[42]	At-risk/ MCI cohort	Significant ↑ in executive function	No improvement	p = 0.049	Small-moderate	Favours VA
Barcelos (2015) [43]	Healthy Older adults	↑Engagement and executive function	No improvement	p = 0.02	Small-moderate	Favours VA
Miki (2014) [54]	Older adults with cancer	↑ Adherence and cognitive function	Stable performance	p < 0.05	Small-moderate	Favours VA
Ridgel (2019)[57]	Older adults with PD	Significant ↑ in cycling efficiency	Significantly ↑ but smaller than VA	VA: p = 0.002; non-VA: p = 0.012	Small (both improved, VA stronger)	Favours VA

Effect size categories inferred as follows: p ≤ 0.001 = large, 0.001 < p ≤ 0.05 = small-moderate, non-significant =trivial/no effect; Standard deviations, and sample sizes unable to report as standardized mean differences(e.g., Hedges’ g) could not be calculated directly [66]

*VA *Virtual aid, *MCI* Mild cognitive impairment, *RCT* Randomized Controlled Trial

Across studies, VA-supported stationary bike interventions were delivered by exercise professionals, researchers, or therapists, with protocols typically involving interactive or videogame-based cycling, cognitive tasks, or prescribed cadence [27, 33, 38, 40–43, 54, 57]. Sessions were most commonly prescribed 2–3 times per week, lasting 30–45 min, over 4–6 weeks or up to 3 months. Interventions were tailored through adjustments to speed, cadence, or HR targets (typically 60–70% HRmax), as well as cognitive challenge scaling *(see Appendix:1 TIDieR framework*,* more detail in PRISMA checklist Appendix:2*).

### Results of syntheses

No meta-analysis was performed due to substantial heterogeneity across the included studies. Possible sources of heterogeneity included differences in study populations (e.g., healthy vs. individuals with chronic diseases), variations in SB intervention types (e.g., ergometers vs. VA-assisted SB), and differing outcome measures and assessment tools. The risk of bias varied, with RCTs generally having a lower risk of bias compared to non-RCTs.

*Key Findings Across Syntheses*:

- SB interventions demonstrated significant improvements in cognitive function, mobility, and cardiovascular fitness, particularly in physically inactive and cognitively impaired individuals.
- VA-assisted SB interventions showed greater benefits for executive function and cycling efficiency compared to traditional SB.
- Aerobic capacity and mobility improvements were more pronounced in healthy individuals compared to those with pre-existing conditions.

Sensitivity analyses were not conducted due to the narrative synthesis approach. However, subgroup analyses suggest that SB interventions, particularly those incorporating VA, provide significant health benefits for older adults, while physically inactive individuals experienced smaller effects on mobility and aerobic capacity.

### Certainty of evidence

The certainty of evidence was assessed considering study quality, risk of bias, and consistency of results.

- RCTs: Generally had lower risk of bias; 16 studies had “good” quality (PEDro score 6–8), while 9 studies scored “fair” (PEDro > 4).
- Non-RCTs: 9 studies had the highest methodological quality (Newcastle-Ottawa Scale score 9), while 5 studies scored between 7 and 8.
- Limitations: Variability in intervention protocols, sample populations, and outcome measures contributed to moderate certainty in the evidence.

## Discussion

This systematic review represents the first comprehensive analysis focusing on the acceptability and effectiveness of SB intervention concerning health outcomes among individuals aged 60 and above. Encompassing 47 intervention studies, including 28 RCTs and 19 non-RCT studies, the review revealed an overall acceptability rate of 38%, retention rate of 15%, adherence rate of 6%, and dropout rate of 13%. Collectively, these studies highlight the wide-ranging positive impacts of SB intervention across multiple domains, including cognitive function, motor skills and balance, physiological and psychological well-being, cardiovascular health, executive function, cycling efficiency, quality of life, musculoskeletal health, as well as feasibility and long-term effectiveness.

According to the current evidence, SB is proposed as a suitable form of endurance training for promoting overall physical health among older adults over 60, primarily due to its relative ease of performance and safety, with no associated injuries reported [7]. Furthermore, the use of a bicycle ergometer in SB presents advantages over treadmill walking, particularly for older frail individuals, as it requires less upper body motion, facilitating the recording of vital signs and blood sample collection [67].

Evidence from SB interventions with VA indicates benefits for both cognition and physical performance. Studies reported significant improvements in executive function (d = 0.5) [39], memory *(*η² = 0.07) [47], walking capacity [49], and cycling efficiency with repeated participation [52]. While these findings highlight the potential of VA-supported cycling, the evidence remains mixed in methodological quality, with several studies relying on non-randomized or pre-post designs, reinforcing the need for further high-quality RCTs to confirm these effects.

Across RCTs, VA-enhanced interventions consistently outperformed non-VA conditions in older adults, improving cycling efficiency, cognition, and executive function. Prior studies confirm that interactive technologies enhance motivation and adherence in aging populations [41, 43]. Benefits were observed in healthy adults, as well as in dementia, diabetes, cancer, and Parkinson’s disease cohorts [33, 38, 40, 42, 54, 57]. Although effect sizes were small to moderate, the consistent direction of benefit underscores.

Additionally, based on RCT evidence it is also found that maximum studies recommended 30 min of SB with VA intervention at ~ 60% HRmax, for 2**–**3 times per week for at least 4**–**6 weeks to achieve significant health benefits (see Appendix: 1). Therefore, SB with VA interventions is a promising tool for promoting physical and cognitive health in late life and require more research.

### Comparison with other studies

Although numerous systematic reviews have explored the benefits of SB for the general population, there is a scarcity of studies focusing specifically on older adults aged 60 and above. Compared with earlier reviews, our study offers a more comprehensive synthesis of stationary bike interventions among older adults (≥ 60 years). Prior work often targeted narrower subgroups or reported heterogeneous protocols, which limited generalizability such as [40] & [33]. For a better example Bouaziz et al. (2015) reviewed cycle ergometer training in adults aged ≥ 70 years but our review extends applicability by including both healthy and clinical populations from 60 years onwards without limiting study types. This broader perspective provides more generalizable and evidence-based recommendations for implementation in both clinical and community settings. Therefore, we advocate for more research into SB cycling among older adults (≥ 60 years) to recommend disease specific exercise dosage to get a better health outcome.

### Strength of the study

This review is robust due to its comprehensive data collection and rigorous methodology. By searching seven multidisciplinary databases and applying broad selection criteria, we captured a wide range of relevant studies. Independent review, multiple testing of data inclusion, and evidence-based acceptability assessments ensured reliability. Study quality was evaluated using the PEDro scale and ROB 2.0 from the Cochrane Handbook, revealing mostly good quality and low risk of bias. Use of the PRISMA checklist and TIDieR framework enhanced transparency and rigor. Focusing on older adults (60+) addressed a neglected population, and including both RCTs and non-RCTs provided a comprehensive understanding of SB interventions, balancing internal validity with real-world applicability.

### Challenges

Included limited access to full-text articles and the lack of a standardized tool for assessing acceptability of SB interventions. Due to sparse data, studies were deemed fully acceptable only with 100% acceptance, adherence, and retention, with no dropouts. Missing data may have affected acceptability estimates, and dropout rates inversely influenced adherence and retention, complicating interpretation. Effect sizes (e.g., Hedges’ g) could not be calculated due to unreported data, so inferred effect sizes were considered [66]. Studies combining SB with VA showed more consistent data than other combinations, highlighting VA as a promising intervention tool. Despite these limitations, the review provides valuable insights into the efficacy of SB interventions in older adults.

## Conclusion

A review of 47 studies found that stationary biking shows moderate acceptability (38%) and yields significant benefits in VO_2_ max, cycling efficiency, cognition, executive function, quality of life, and mobility (Timed Up and Go test) in adults aged ≥ 60. When combined with VA, SB improves adherence and further enhances physical and cognitive outcomes across both healthy and clinical populations. Therefore, high quality RCTs are needed to establish optimal exercise prescriptions, and to support stronger evidence for SB combined with VA as a promising, low-risk strategy to promote healthy aging and maintain functional independence in later life.

## Supplementary Information

Supplementary Material 1.

Supplementary Material 2.

Supplementary Material 3.

Supplementary Material 4.

## Data Availability

This review did not utilize primary data. Refer to the cited paper for primary data sources. All data in this study are publicly accessible and previously published. The compiled dataset analyzed in this study can be obtained from the corresponding author upon reasonable request.
