# Supplementary material for: Acceptability and effectiveness of stationary bike intervention on health outcomes among older adults: a systematic review of intervention studies
Source: BMC Geriatr. 2026 Jan 13;26:130. doi: 10.1186/s12877-025-06757-0 (PMC12857070; doi:10.1186/s12877-025-06757-0)
Supplement: Supplementary file 1 — Supplementary Material 1. [file 12877_2025_6757_MOESM1_ESM.docx]

**Supplementary table 1.** Different health outcomes after stationary bike intervention

| **First Author**  **(Year of publication))** | **health outcomes after stationary bike intervention** | | | | | | | | | | | | | | | | |
| --- | --- | --- | --- | --- | --- | --- | --- | --- | --- | --- | --- | --- | --- | --- | --- | --- | --- |
|  | Executive function 5 | | Cognition | | Memory | Aerobic capacity (VO2  Max) | Cycling efficiency | | Quality Of Life (QOL) | Balance (BBS test) | Mobility (TUG  test) | Walking Capacity | Gait | Pain reduction | Lowering of BP | Lowering of HR |  |
|  | Colour trails test | Stroop a/c test | CR time (↑) (not improved) | CR time (↓) (improved) |  |  | With VA | Without VA |  |  |  |  |  |  |  |  |  |
| Abbas (2022) |  |  |  |  |  |  |  |  |  | + | (-) |  |  |  |  |  |  |
|  |  |  |  |  |  |  |  |  |  | *(p<0.05)* | *(p<0.05)* |  |  |  |  |  |  |
| Anderson- | + |  |  |  |  |  |  |  |  |  |  |  |  |  |  |  |  |
| Hanley, C. | *(p=0.002* |  |  |  |  |  |  |  |  |  |  |  |  |  |  |  |  |
| (2012) | *)* |  |  |  |  |  |  |  |  |  |  |  |  |  |  |  |  |
| Anderson- | + |  |  |  |  |  |  |  |  |  |  |  |  |  |  |  |  |
| Hanley | *(p=0.007* |  |  |  |  |  |  |  |  |  |  |  |  |  |  |  |  |
| (2012) | *)* |  |  |  |  |  |  |  |  |  |  |  |  |  |  |  |  |
| Anderson- |  | + |  |  |  |  |  |  |  |  |  |  |  |  |  |  |  |
| Hanley |  | *(p=0.049* |  |  |  |  |  |  |  |  |  |  |  |  |  |  |  |
| (2018) |  | *)* |  |  |  |  |  |  |  |  |  |  |  |  |  |  |  |
| Anderson- |  |  |  |  |  |  | + |  |  |  |  |  |  |  |  |  |  |
| Hanley |  |  |  |  |  |  | *(p=0.003* |  |  |  |  |  |  |  |  |  |  |
| (2011) |  |  |  |  |  |  | *)* |  |  |  |  |  |  |  |  |  |  |
|  |  |  |  |  |  |  | *(↑Compet* |  |  |  |  |  |  |  |  |  |  |
|  |  |  |  |  |  |  | *itiveness* |  |  |  |  |  |  |  |  |  |  |
|  |  |  |  |  |  |  | *and* |  |  |  |  |  |  |  |  |  |  |
|  |  |  |  |  |  |  | *exercise* |  |  |  |  |  |  |  |  |  |  |
|  |  |  |  |  |  |  | *effort))* |  |  |  |  |  |  |  |  |  |  |
| Antunes |  |  |  |  | + | + |  |  |  |  |  |  |  |  |  |  |  |
| (2015) |  |  |  |  | *(p <0.05)* | *(p <0.05)* |  |  |  |  |  |  |  |  |  |  |  |
| Barcelos | + | (-) |  |  |  |  |  |  |  |  |  |  |  |  |  |  |  |
| (2015) | *(p=0.02)* |  |  |  |  |  |  |  |  |  |  |  |  |  |  |  |  |

| Bellumori |  |  |  |  |  |  |  |  |  |  | + |  |  |  |  |  |
| --- | --- | --- | --- | --- | --- | --- | --- | --- | --- | --- | --- | --- | --- | --- | --- | --- |
| (2017) |  |  |  |  |  |  |  |  |  |  | *(p <0.05)* |  |  |  |  |  |
| Briswalter |  |  |  |  |  |  |  | (-) |  |  |  |  |  |  |  |  |
| (2014) |  |  |  |  |  |  |  | *(p* |  |  |  |  |  |  |  |  |
|  |  |  |  |  |  |  |  | *<0.05)* |  |  |  |  |  |  |  |  |
| Buccola |  |  |  |  |  | + |  |  |  |  |  |  |  |  | **+** |  |
| (1975) |  |  |  |  |  | *(p <0.05)* |  |  |  |  |  |  |  |  | *(p <0.05)* |  |
| Callow (2022) |  |  |  |  |  |  |  |  |  |  |  |  |  |  |  |  |
| Cicek (2020) |  |  |  |  |  |  |  |  |  | +  *(p=0.002)* | (-) |  |  |  |  |  |
| Colombo |  |  |  |  |  |  |  |  |  |  |  | *+* |  |  |  |  |
| (2023) |  |  |  |  |  |  |  |  |  |  |  | *(p<0.05)* |  |  |  |  |
| Cunha (2021) |  |  |  |  |  |  |  |  |  |  |  |  |  |  | (-) |  |
| D'Cunha |  |  |  |  |  |  | *(-)* |  |  |  |  |  |  |  |  |  |
| (2021) |  |  |  |  |  |  | *(p=0.012* |  |  |  |  |  |  |  |  |  |
|  |  |  |  |  |  |  | *)* |  |  |  |  |  |  |  |  |  |
|  |  |  |  |  |  |  | *(Challeng* |  |  |  |  |  |  |  |  |  |
|  |  |  |  |  |  |  | *ing and* |  |  |  |  |  |  |  |  |  |
|  |  |  |  |  |  |  | *immersiv* |  |  |  |  |  |  |  |  |  |
|  |  |  |  |  |  |  | *e)* |  |  |  |  |  |  |  |  |  |
| Emery |  |  |  |  |  | ++ |  |  |  |  |  |  |  |  |  |  |
| (1994) |  |  |  |  |  | *(p <0.001)* |  |  |  |  |  |  |  |  |  |  |
| Ferrai (2004) |  |  |  |  |  | ++ |  |  | + |  |  |  |  |  |  |  |
|  |  |  |  |  |  | *(p=0.001****)*** |  |  |  |  |  |  |  |  |  |  |
|  |  |  |  |  |  |  |  |  | *(QOL* |  |  |  |  |  |  |  |
|  |  |  |  |  |  |  |  |  | *score:* |  |  |  |  |  |  |  |
|  |  |  |  |  |  |  |  |  | *7/9, p* |  |  |  |  |  |  |  |
|  |  |  |  |  |  |  |  |  | *value* |  |  |  |  |  |  |  |
|  |  |  |  |  |  |  |  |  | *did not* |  |  |  |  |  |  |  |
|  |  |  |  |  |  |  |  |  | *mention* |  |  |  |  |  |  |  |
|  |  |  |  |  |  |  |  |  | *)* |  |  |  |  |  |  |  |
| Ferraz (2018) |  |  |  |  |  |  |  |  |  |  |  | *++ (p=0.001)* |  |  |  |  |
| Gitlin (1992) |  |  |  |  |  |  |  |  | *++ (p<0.00 1)* |  |  |  |  |  |  |  |
| Hill (2015) |  |  |  |  |  |  |  |  |  |  |  |  | (-) |  |  |  |
| Hou (2023) |  |  |  |  | (+)  *(p=0.021*  ***)*** |  |  |  |  |  |  |  |  |  |  |  |
| Joyce (2014) |  |  |  | *++ (p<0.001)* |  |  |  |  |  |  |  |  |  |  |  |  |
| Katyal (2003) |  |  |  |  |  | +  *(p <0.05)* |  |  |  |  |  |  |  |  |  |  |
| Kwan (2021) |  |  | +  *(p=0.01)*  *(Overall cognitive function)* | |  |  |  |  |  |  |  |  |  |  |  |  |
| Lebeau (2020) |  | *++ (P=0.001*  *)* |  |  |  |  |  |  |  |  |  |  |  |  |  |  |
| Lee (2014) |  |  |  |  |  |  |  |  |  | +  *(p<0.05)* |  |  |  |  |  |  |
| Loggia (2021) |  |  |  |  |  |  | +  *(p <0.05)*  *(Repetitiv e participat ion)* |  |  |  |  |  |  |  |  |  |
| Lopez-Garcia (2019) |  |  | ++  *(p<0.001)* |  |  |  |  |  |  |  |  |  |  |  |  |  |
| Madden (2009) |  |  |  |  |  | (-) |  |  |  |  |  |  |  |  |  |  |
| Mahajan (2021) |  |  |  | |  |  |  | *+*  *(p <0.05* |  |  |  |  |  |  |  |  |

|  |  |  |  | |  |  |  | *(Feasibl e for sleep hygiene and interval Training*  *)* |  |  |  |  |  |  |  |  |
| --- | --- | --- | --- | --- | --- | --- | --- | --- | --- | --- | --- | --- | --- | --- | --- | --- |
| Miki (2014 |  |  | +  *(p=0.006)*  *(Overall cognitive function)* | |  |  |  |  |  |  |  |  |  |  |  |  |
| Morita (2013) |  |  |  |  |  |  |  |  |  |  |  |  |  |  | +  *(p <0.05)*  *(Women)* |  |
| Nocera (2020) |  |  |  |  |  | ++  *(p <0.001)* |  |  |  |  | +  *(p <0.03)* |  |  |  |  |  |
| Pauwels (2018) |  |  |  |  |  |  |  |  |  |  |  |  |  | +  *(p=0.01)* |  |  |
| Posner (1992) |  |  |  |  |  | ++  *(p <0.001)* |  |  |  |  |  |  |  |  |  |  |
| Rezasoltani (2020) |  |  |  |  |  |  |  |  |  |  |  |  |  | ++  *(p*  *<0.001)* |  |  |
| Ridgel (2019) |  |  |  |  |  |  |  |  |  |  | +  *(p=0.002)* |  | +  *(p=0.012)* |  |  |  |
| Salisbury (2022) |  |  |  |  |  | (+)  *(p=0.03****)*** |  |  |  |  |  |  |  |  |  |  |
| Schwarck (2021) |  |  |  | +  *(Significan t but p*  *value was absent))* |  |  |  |  |  |  |  |  |  |  |  |  |
| Tollár (2019) |  |  |  |  |  |  |  |  | (-) | (-) |  | (-) |  |  |  |  |

| VanRoie (2017) |  |  |  |  |  |  |  |  | ++ *(p<0.00 1)* |  |  |  |  |  |  |  |
| --- | --- | --- | --- | --- | --- | --- | --- | --- | --- | --- | --- | --- | --- | --- | --- | --- |
| Willenheimer (1998) |  |  |  |  |  | (-) |  |  | +  *(p<0.01*  *)*  *(Men)* |  |  |  |  |  |  | (-)  *No change* |
| Wu (2023) |  |  | +  *(p≤0.05)* |  |  |  |  |  |  |  |  |  |  |  |  |  |
| Yu (2011) |  |  |  |  |  |  |  |  |  |  |  |  |  |  |  | +  *(p=0.01)* |
| Yu (2021) | (-)  (*Overall executive function)* | | (-)  (*Overall cognitive function)* | | (-) |  |  |  |  |  |  |  |  |  |  |  |

*N=45 studies tabulated out of 47 studies*

*Here p-value considered as statistically significant (+) if, p is ≤ 0.05; highly significant (++) if, p is ≤0.001; and not significant if, p is > 0.05. Minus (-) signifies: Stationary bikes intervention failed to show any better improvement over other intervention among the study or trial group.*

*CR: Choice Reaction, VA: Virtual Aid, BP: Blood pressure, HR: Heart rate, BBS: Berg Balance Scale, TUG: Timed Up & Go, VO2 Max: Maximum oxygen capacity.*

**Supplementary table 2.** Assessment of study quality of the 28 Randomised Control Trials s according to PEDro scale

| **Study** | **Criterion 02: Random allocation** | **Criterion 03: Concealed allocation** | **Criterion 04:**  **Groups similar at baseline** | **Criterion 05: Participant  blinding** | **Criterion 06: Therapist  blinding** | **Criterion 07: Assessor blinding** | **Criterion 08:**  **more than 85% of outcomes measured** | **Criterion 09: Intention-to-treat analysis** | **Criterion 10: Between-group  difference  reported** | **Criterion 11:**  **Point estimate  and variability  reported** | **Total (0 to 10)** |
| --- | --- | --- | --- | --- | --- | --- | --- | --- | --- | --- | --- |
| Abbas et al. 2022 | Y | N | Y | N | N | Y | Y | Y | Y | Y | 7 |
| Anderson-Hanley et al. 2012 | Y | Y | Y | N | N | N | Y | Y | Y | Y | 6 |
| Anderson-Hanley et al. 2011 | Y | N | Y | N | N | N | Y | N | Y | Y | 5 |
| Anderson-Hanley et al. 2018 | Y | N | Y | N | N | N | Y | N | Y | Y | 5 |
| Antunes et al. 2015 | Y | N | Y | N | N | N | Y | N | Y | Y | 5 |
| Barcelos et al. 2015 | Y | N | N | N | N | N | Y | N | Y | Y | 4 |
| Bellumori et al. 2017 | Y | N | Y | N | N | N | Y | N | Y | Y | 5 |
| D’Cunha et al. 2021 | Y | Y | N | N | N | N | Y | N | Y | Y | 4 |
| Ferraz et al. 2018 | Y | Y | Y | N | N | Y | Y | N | Y | Y | 7 |
| Gitlin et al. 1992 | Y | N | Y | N | N | N | Y | Y | Y | Y | 6 |
| Hou et al. 2023 | Y | N | Y | N | N | Y | Y | N | Y | Y | 6 |
| Kwan et al. 2021 | Y | N | N | N | Y | Y | Y | Y | Y | Y | 7 |
| Lebeau et al. 2020 | Y | N | N | N | N | N | Y | Y | Y | Y | 5 |
| Lo´ pez-Garcı´a et al. 2019 | Y | N | N | N | N | N | Y | Y | Y | Y | 5 |
| Madden et al. 2009 | Y | Y | Y | N | N | Y | Y | Y | Y | Y | 8 |
| Mahajan et al. 2021 | Y | Y | Y | Y | Y | Y | Y | Y | Y | Y | 10 |
| Miki et al. 2014 | Y | Y | Y | N | N | Y | Y | N | Y | Y | 7 |
| Morita et al. 2013 | Y | N | N | N | N | N | Y | Y | Y | Y | 5 |
| Nocera et al. 2020 | Y | Y | Y | Y | N | N | Y | Y | Y | Y | 8 |
| Posner et al. 1992 | Y | N | N | N | N | N | Y | Y | Y | Y | 5 |
| Rezasoltani et al. 2020 | Y | Y | Y | N | Y | Y | Y | Y | Y | Y | 9 |
| Ridgel et al. 2019 | Y | N | Y | N | N | N | Y | Y | Y | N | 5 |
| Salisbury ey al. 2022 | Y | Y | N | Y | Y | N | Y | Y | Y | Y | 8 |
| Tollar et al. 2019 | Y | N | Y | N | N | N | Y | N | Y | Y | 5 |
| Roie et al. 2017 | Y | Y | N | Y | N | Y | Y | N | Y | Y | 7 |
| Willenheimer et al. 1998 | Y | N | N | N | N | N | Y | Y | Y | Y | 5 |
| Wu et al. 2023 | Y | N | Y | N | N | Y | Y | N | Y | Y | 6 |
| Yu et al. 2023 | Y | Y | N | N | N | Y | Y | Y | Y | Y | 7 |

**Supplementary table 3.** Risk of Bias assessment of 28 Randomised Controlled Trials according to the Cochrane risk of bias tool, version 2

| Criterion | Randomization process | Deviations from intended interventions | Missing outcome data | Measurement of the outcome | Selection of the reported result | Overall |
| --- | --- | --- | --- | --- | --- | --- |
|  |  |  |  |  |  |  |
| Abbas et al. 2022 | 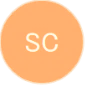 | 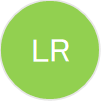 | 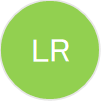 | 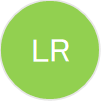 | 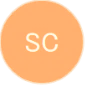 | 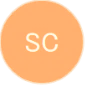 |
| Anderson-Hanley et al. 2012 | 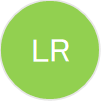 | 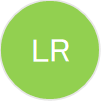 | 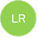 | 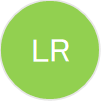 | 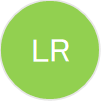 | 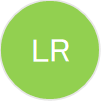 |
| Anderson-Hanley et al. 2011 | 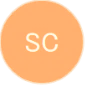 | 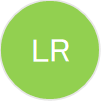 | 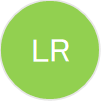 | 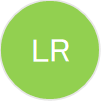 | 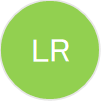 | 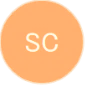 |
| Anderson-Hanley et al. 2018 | 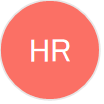 | 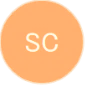 | 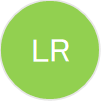 | 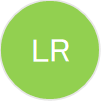 | 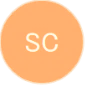 | 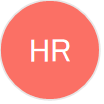 |
| Antunes et al. 2015 | 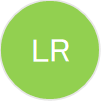 | 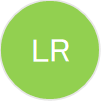 | 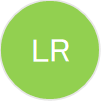 | 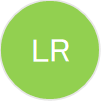 | 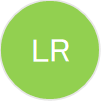 | 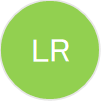 |
| Barcelos et al. 2015 | 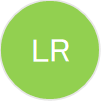 | 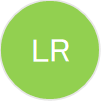 | 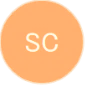 | 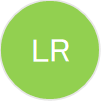 | 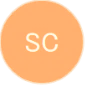 | 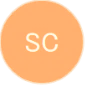 |
| Bellumori et al. 2017 | 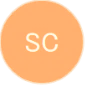 | 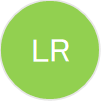 | 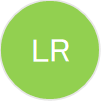 | 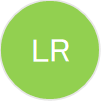 | 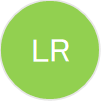 | 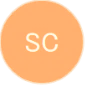 |
| D’Cunha et al. 2011 | 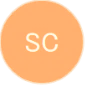 | 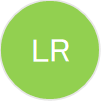 | 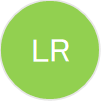 | 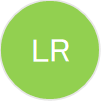 | 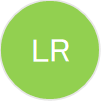 | 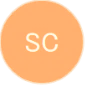 |
| Ferraz et al. 2018 | 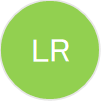 | 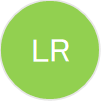 | 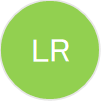 | 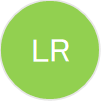 | 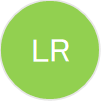 | 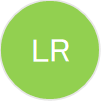 |
| Gitlin et al. 1992 | 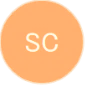 | 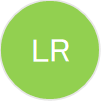 | 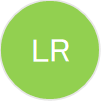 | 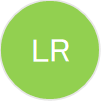 | 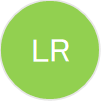 | 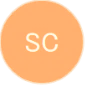 |
| Hou et al. 2023 | 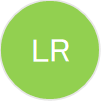 | 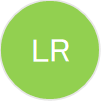 | 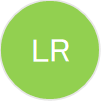 | 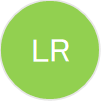 | 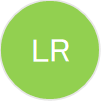 | 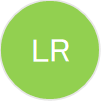 |
| Kwan et al. 2021 | 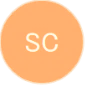 | 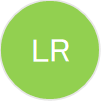 | 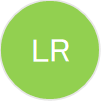 | 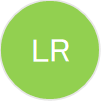 | 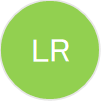 | 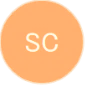 |
| Lebeau et al. 2020 | 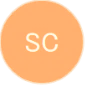 | 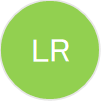 | 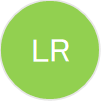 | 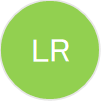 | 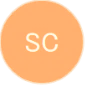 | 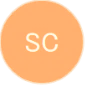 |
| Lo´ pez-Garcı´a et al. 2019 | 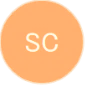 | 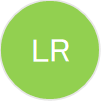 | 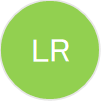 | 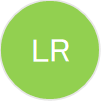 | 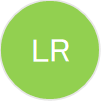 | 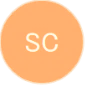 |
| Madden et al. 2009 | 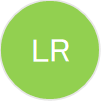 | 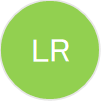 | 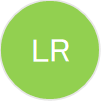 | 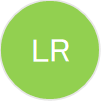 | 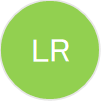 | 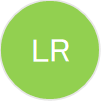 |
| Mahajan et al. 2021 | 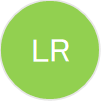 | 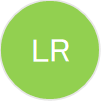 | 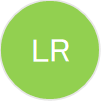 | 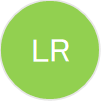 | 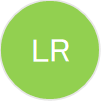 | 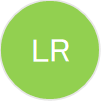 |
| Miki et al. 2014 | 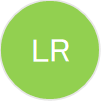 | 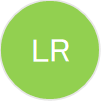 | 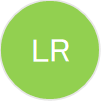 | 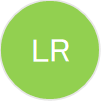 |  |  |
| Morita et al. 2013 |  |  |  |  |  |  |
| Nocera et al. 2020 |  |  |  |  |  |  |
| Posner et al. 1992 |  |  |  |  |  |  |
| Rezasoltani et al. 2020 |  |  |  |  |  |  |
| Ridgel et al. 2019 |  |  |  |  |  |  |
| Salisbury et al. 2022 |  |  |  |  |  |  |
| Tollar et al. 2019 |  |  |  |  |  |  |
| Roie et al. 2017 |  |  |  |  |  |  |
| Willenheimer et al. 1998 |  |  |  |  |  |  |
| Wu et al. 2023 |  |  |  |  |  |  |
| Yu et al. 2023 |  |  |  |  |  |  |

**Supplementary table 4.** Quality assessment of the 19 non-randomized controlled trials according to Newcastle-Ottawa Scale

| **Study (year)** | **Selection** | | | | **Comparability** | **Outcome** | | | **Score** |
| --- | --- | --- | --- | --- | --- | --- | --- | --- | --- |
|  | Representativeness of the exposed cohort | Selection of the non- exposed cohort | Ascertainment of exposure | Outcome of interest was not present at start of  study | Comparability of cohorts on the basis of the design or  analysis | Assessment of outcome | Adequate follow-up duration | Adequate follow-up rate |  |
| **1. Anderson (2012)** | ★ | ★ | - | ★ | ★ | ★ | ★ | ★ | 7 |
| **2. Brisswalter (2014)** | ★ | ★ | ★ | - | ★ | ★ | ★ | ★ | 7 |
| **3. Buccola (1975)** | - | - | - | ★ | ★ | ★ | ★ | ★ | 5 |
| **4. Callow (2022)** | ★ | ★ | ★ | ★ | ★★ | ★ | ★ | ★ | 9 |
| **5. Cicek (2020)** | ★ | ★ | ★ | ★ | ★★ | ★ | ★ | ★ | 9 |
| **6. Colombo (2023)** | ★ | ★ | ★ | ★ | ★★ | ★ | ★ | ★ | 9 |
| **7. Cunha (2021)** | ★ | ★ | ★ | ★ | ★★ | ★ | ★ | ★ | 9 |
| **8. Emery (1994)** | ★ | ★ | ★ | ★ | ★ | ★ | ★ | ★ | 8 |
| **9. Ferrai (2004)** | ★ | ★ | - | ★ | ★★ | ★ | ★ | ★ | 8 |
| **10. Gaesser (2018)** | ★ | ★ | ★ | - | ★ | ★ | ★ | ★ | 7 |
| **11. Hill (2015)** | ★ | ★ | ★ | ★ | ★★ | ★ | ★ | ★ | 9 |
| **12. Joyce (2014)** | ★ | ★ | ★ | ★ | ★★ | ★ | ★ | ★ | 9 |
| **13. Katyal (2003)** | ★ | ★ | ★ | ★ | ★★ | ★ | ★ | ★ | 9 |
| **14. Lee (2014)** | ★ | ★ | ★ | ★ | ★ | ★ | ★ | ★ | 8 |
| **15. Loggia (2021)** | ★ | ★ | ★ | ★ | ★ | ★ | ★ | ★ | 8 |
| **16. Pauwels (2018)** | - | ★ | ★ | ★ | ★★ | ★ | - | ★ | 7 |
| **17. Schwarck (2021)** | ★ | ★ | ★ | ★ | ★★ | ★ | ★ | ★ | 9 |
| **18. Yu (2011)** | ★ | ★ | ★ | ★ | ★★ | ★ | ★ | ★ | 9 |
| **19. Yu (2013)** | ★ | **-** | ★ | **-** | **-** | **-** | **-** | ★ | 3 |

44

**Supplementary Figure 1.** Trend of publication

9

8

7

6

5

4

3

2

1

0

1975-1980 1981-1986 1987-1992 1993-1998 1999-2004 2005-2010 2011-2016 2017-2023

Cognitive function

Motor and Balance

Physiological and psychological changes

Cardiovascular system

Cycling efficiency and QOL Executive function

Musculoskeletal VR induced training Feasibility and effectiveness

*QOL=Quality of life, VR=Virtual reality*

**References;**

**Randomized controlled trials:**

**[1–28]**

1. Abbas RL, Saab IM, Al-Sharif HK, Naja N, El-Khatib A. Effect of Adding Motorized Cycle Ergometer Over Exercise Training on Balance in Older Adults with Dementia: A Randomized Controlled Trial. Experimental Aging Research. 2023;49:100–11.

2. Anderson-Hanley C, Arciero PJ, Brickman AM, Nimon JP, Okuma N, Westen SC, et al. Exergaming and Older Adult Cognition. American Journal of Preventive Medicine. 2012;42:109–19.

3. Anderson-Hanley C, Arciero P, Snyder. Social facilitation in virtual reality-enhanced exercise: competitiveness moderates exercise effort of older adults. CIA. 2011;:275.

4. Anderson-Hanley C, Barcelos NM, Zimmerman EA, Gillen RW, Dunnam M, Cohen BD, et al. The Aerobic and Cognitive Exercise Study (ACES) for Community-Dwelling Older Adults With or At-Risk for Mild Cognitive Impairment (MCI): Neuropsychological, Neurobiological and Neuroimaging Outcomes of a Randomized Clinical Trial. Front Aging Neurosci. 2018;10:76.

5. Antunes HK, De Mello MT, Santos-Galduróz RF, Galduróz JCF, Lemos VA, Tufik S, et al. Effects of a physical fitness program on memory and blood viscosity in sedentary elderly men. Braz J Med Biol Res. 2015;48:805–12.

6. Barcelos N, Shah N, Cohen K, Hogan MJ, Mulkerrin E, Arciero PJ, et al. Aerobic and Cognitive Exercise (ACE) Pilot Study for Older Adults: Executive Function Improves with Cognitive Challenge While Exergaming. J Int Neuropsychol Soc. 2015;21:768–79.

7. Bellumori M, Uygur M, Knight CA. High-Speed Cycling Intervention Improves Rate-Dependent Mobility in Older Adults. Medicine & Science in Sports & Exercise. 2017;49:106–14.

8. D’Cunha NM, Isbel ST, Frost J, Fearon A, McKune AJ, Naumovski N, et al. Effects of a virtual group cycling experience on people living with dementia: A mixed method pilot study. Dementia. 2021;20:1518–35.

9. Ferraz DD, Trippo KV, Duarte GP, Neto MG, Bernardes Santos KO, Filho JO. The Effects of Functional Training, Bicycle Exercise, and Exergaming on Walking Capacity of Elderly Patients With Parkinson Disease: A Pilot Randomized Controlled Single-blinded Trial. Archives of Physical Medicine and Rehabilitation. 2018;99:826–33.

10. Gitlin LN, Lawton MP, Windsor-landsberg lisa A, Kleban MH, Sands laura P, Posner J. In search of Psychological benefits exercise in healthy older adults. Journal of agining and health. 1992;4:174–92.

11. Hou H-Y, Chen J, Hai L, Wang P, Zhang J-X, Li H-J. Effects of exergame and bicycle exercise intervention on blood pressure and executive function in older adults with hypertension: A three-group randomized controlled study. Experimental Gerontology. 2023;173:112099.

12. Kwan RYC, Liu JYW, Fong KNK, Qin J, Leung PK-Y, Sin OSK, et al. Feasibility and Effects of Virtual Reality Motor-Cognitive Training in Community-Dwelling Older People With Cognitive Frailty: Pilot Randomized Controlled Trial. JMIR Serious Games. 2021;9:e28400.

13. Lebeau J-C, Mason J, Roque N, Tenenbaum G. The effects of acute exercise on driving and executive functions in healthy older adults. International Journal of Sport and Exercise Psychology. 2022;20:283–301.

14. López-García J, Colado JC, Guzmán JF. Acute Effects of Aerobic Exercise and Active Videogames on Cognitive Flexibility, Reaction Time, and Perceived Exertion in Older Adults. Games for Health Journal. 2019;8:371–9.

15. Madden KM, Lockhart C, Cuff D, Potter TF, Meneilly GS. Short-Term Aerobic Exercise Reduces Arterial Stiffness in Older Adults With Type 2 Diabetes, Hypertension, and Hypercholesterolemia. Diabetes Care. 2009;32:1531–5.

16. Mahajan A, Mahajan S, Tilekar S. A Pilot Randomized Controlled Trial of Interval Training and Sleep Hygiene for Improving Sleep in Older Adults. Journal of Aging and Physical Activity. 2021;29:993–1002.

17. Miki E, Kataoka T, Okamura H. Feasibility and efficacy of speed-feedback therapy with a bicycle ergometer on cognitive function in elderly cancer patients in Japan: Speed-feedback therapy in elderly cancer patients. Psycho-Oncology. 2014;23:906–13.

18. Morita N, Okita K. Is Gender a Factor in the Reduction of Cardiovascular Risks With Exercise Training? Circ J. 2013;77:646–51.

19. Nocera JR, Mammino K, Kommula Y, Wharton W, Crosson B, McGregor KM. Effects of Combined Aerobic Exercise and Cognitive Training on Verbal Fluency in Older Adults. Gerontology and Geriatric Medicine. 2020;6:233372141989688.

20. Posner JD, Gorman KM, Windsor‐Landsberg L, Larsen J, Bleiman M, Shaw C, et al. Low to Moderate Intensity Endurance Training in Healthy Older Adults: Physiological Responses after Four Months. J American Geriatrics Society. 1992;40:1–7.

21. Rezasoltani Z, Sanati E, Kazempour Mofrad R, Azizi S, Dadarkhah A, Najafi S. Randomized Controlled Trial of Aquatic Cycling for Treatment of Knee Osteoarthritis in Elderly People. Topics in Geriatric Rehabilitation. 2020;36:103–9.

22. Ridgel AL, Ault DL. High-Cadence Cycling Promotes Sustained Improvement in Bradykinesia, Rigidity, and Mobility in Individuals with Mild-Moderate Parkinson’s Disease. Parkinson’s Disease. 2019;2019:1–7.

23. Salisbury D, Mathiason MA, Yu F. Exercise Dose and Aerobic Fitness Response in Alzheimerʼs Dementia: Findings from the FIT-AD Trial. Int J Sports Med. 2022;43:850–8.

24. Tollár J, Nagy F, Moizs M, Tóth BE, Sanders LMJ, Hortobágyi T. Diverse Exercises Similarly Reduce Older Adults’ Mobility Limitations. Medicine & Science in Sports & Exercise. 2019;51:1809–16.

25. Van Roie E, Martien S, Hurkmans E, Pelssers J, Seghers J, Boen F, et al. Ergometer-cycling with strict versus minimal contact supervision among the oldest adults: A cluster-randomised controlled trial. Archives of Gerontology and Geriatrics. 2017;70:112–22.

26. Willenheimer R. Exercise training in heart failure improves quality of life and exercise capacity. European Heart Journal. 1998;19:774–81.

27. Wu S, Ji H, Won J, Jo E-A, Kim Y-S, Park J-J. The Effects of Exergaming on Executive and Physical Functions in Older Adults With Dementia: Randomized Controlled Trial. J Med Internet Res. 2023;25:e39993.

28. Yu F, Vock DM, Zhang L, Salisbury D, Nelson NW, Chow LS, et al. Cognitive Effects of Aerobic Exercise in Alzheimer’s Disease: A Pilot Randomized Controlled Trial. JAD. 2021;80:233–44.

**Non-randomized controlled trials:**

**[1–19]**

1. Anderson-Hanley C, Arciero PJ, Westen SC, Nimon J, Zimmerman E. Neuropsychological Benefits of Stationary Bike Exercise and a Cybercycle Exergame for Older Adults with Diabetes: An Exploratory Analysis. J Diabetes Sci Technol. 2012;6:849–57.

2. Brisswalter J, Wu SSX, Sultana F, Bernard T, Abbiss CR. Age difference in efficiency of locomotion and maximal power output in well-trained triathletes. Eur J Appl Physiol. 2014;114:2579–86.

3. Buccola VA, Stone WJ. Effects of Jogging and Cycling Programs on Physiological and Personality Variables in Aged Men. Research Quarterly American Alliance for Health, Physical Education and Recreation. 1975;46:134–9.

4. Callow DD, Pena GS, Stark CEL, Smith JC. Effects of acute aerobic exercise on mnemonic discrimination performance in older adults. J Int Neuropsychol Soc. 2023;29:519–28.

5. Cicek A, Ozdincler AR, Tarakci E. Interactive video game-based approaches improve mobility and mood in older adults: A nonrandomized, controlled tri̇al. Journal of Bodywork and Movement Therapies. 2020;24:252–9.

6. Colombo V, Mondellini M, Fumagalli A, Aliverti A, Sacco M. A virtual reality-based endurance training program for COPD patients: acceptability and user experience. Disability and Rehabilitation: Assistive Technology. 2023;:1–10.

7. Cunha RM, Arsa G, Oliveira-Silva I, Ferreira Rocha I, Machado Lehnen A. Acute Blood Pressure Effects in Older Adults with Hypertension After Different Modalities of Exercise: An Experimental Study. Journal of Aging and Physical Activity. 2021;29:952–8.

8. Emery CF. Effect of age on physiological and psychological functioning among COPD patients in an exercise program. Journal of agining and health. 1994;6:3–16.

9. Ferrari M, Vangelista A, Vedovi E, Falso M, Segattini C, Brotto E, et al. Minimally Supervised Home Rehabilitation Improves Exercise Capacity and Health Status in Patients with COPD: American Journal of Physical Medicine & Rehabilitation. 2004;83:337–43.

10. Gaesser GA, Tucker WJ, Sawyer BJ, Bhammar DM, Angadi SS. Cycling efficiency and energy cost of walking in young and older adults. Journal of Applied Physiology. 2018;124:414–20.

11. Hill MW, Oxford SW, Duncan MJ, Price MJ. The effects of arm crank ergometry, cycle ergometry and treadmill walking on postural sway in healthy older females. Gait & Posture. 2015;41:252–7.

12. Joyce J, Smyth PJ, Donnelly AE, Davranche K. The Simon Task and Aging: Does Acute Moderate Exercise Influence Cognitive Control? Medicine & Science in Sports & Exercise. 2014;46:630–9.

13. Katyal S, Freeman M, Miller JA, Thomas SG. Short-term aerobic training and circulatory function in women: age and hormone- replacement therapy. 2003.

14. Lee C-W, Cho G-H. Effect of Stationary Cycle Exercise on Gait and Balance of Elderly Women. J Phys Ther Sci. 2014;26:431–3.

15. Loggia G, Gauthier A, Lemiere F, Drigny J, Desvergee A, Leconte P, et al. Cycle more with virtual reality: a proof of concept study in an institutionalised able-bodied geriatric population. Age and Ageing. 2021;50:1422–5.

16. Pauwels C, Roren A, Gautier A, Linières J, Rannou F, Poiraudeau S, et al. Home-based cycling program tailored to older people with lumbar spinal stenosis: Barriers and facilitators. Annals of Physical and Rehabilitation Medicine. 2018;61:144–50.

17. Schwarck S, Busse N, Ziegler G, Glanz W, Becke A, Düzel E. Heart Rate Variability During Physical Exercise Is Associated With Improved Cognitive Performance in Alzheimer’s Dementia Patients—A Longitudinal Feasibility Study. Front Sports Act Living. 2021;3:684089.

18. Yu F. Improving recruitment, retention, and adherence to 6-month cycling in Alzheimer’s disease. Geriatric Nursing. 2013;34:181–6.

19. Yu F, Savik K, Wyman JF, Bronas UG. Maintaining Physical Fitness and Function in Alzheimer’s Disease: A Pilot Study. Am J Alzheimers Dis Other Demen. 2011;26:406–12.
