# Supplementary material for: Acceptability and effectiveness of stationary bike intervention on health outcomes among older adults: a systematic review of intervention studies
Source: BMC Geriatr. 2026 Jan 13;26:130. doi: 10.1186/s12877-025-06757-0 (PMC12857070; doi:10.1186/s12877-025-06757-0)
Supplement: Supplementary file 2 — Supplementary Material 2. [file 12877_2025_6757_MOESM2_ESM.pdf]

Search string: [“old people” or “older people” or “elderly” or “elders” or “aging” or “ageing” or “old men” or “old women” or “older persons” or “older adults” or “seniors”] (AND) [“bicycling” or “cycling” or “biking” or “bike” or “bicycle”]

Timeline: Articles Published until 22nd December 2021		
Databases	Number of Hits	No. of relevant articles
PubMed	70	06
Web of Science	103	04
Scopus	541	06
Cochrane library	408	07
SportDiscus	60	07
CINAHL	45	03
PsychInfo	Not found	00
Total Hits	1227	33
Duplicates	81	
Timeline: Articles published from 23rd December 2021 to 18th June 2023 / “or” Dec 2021 to June 2023		
Databases	Number of Hits	No. of relevant articles
PubMed	471	444
Web of Science	337	310
Scopus	2189	2163
Cochrane library	3027	2999
SportDiscus	410	383
CINAHL	181	154
PsychInfo	180	153
Total number	6795	6606
Duplicates	1302	
**Grand total hits**	**6795+1731=8022**	
**Grand total duplicates**	**1302+81=1383**	
**Grand total number of relevant articles**	**6606+33=6639**	

Evidence of searches in different Databases

Published until 22nd December 2021

PUBMED:

SearchActionsDetailsQueryResultsTime#5
Search: **((((((((((("old people"[Title/Abstract]) OR ("older people"[Title/Abstract])) OR ("elderly"[Title/Abstract])) OR ("elders"[Title/Abstract])) OR ("aging"[Title/Abstract])) OR ("ageing"[Title/Abstract])) OR ("old men"[Title/Abstract])) OR ("old women"[Title/Abstract])) OR ("older persons"[Title/Abstract])) OR ("older adults"[Title/Abstract])) OR ("seniors"[Title/Abstract])) AND ((("bicycling"[Title/Abstract]) AND ("cycling"[Title/Abstract])) OR (((("bicycling"[Title/Abstract]) OR ("biking"[Title/Abstract])) OR ("bike"[Title/Abstract])) OR ("bicycle"[Title/Abstract])))** Sort by: **Most Recent**
47116:46:24#4

Search: **(("bicycling"[Title/Abstract]) AND ("cycling"[Title/Abstract])) OR (((("bicycling"[Title/Abstract]) OR ("biking"[Title/Abstract])) OR ("bike"[Title/Abstract])) OR ("bicycle"[Title/Abstract]))** Sort by: **Most Recent**
15,97912:38:39#3

Search: **((("bicycling"[Title/Abstract]) OR ("biking"[Title/Abstract])) OR ("bike"[Title/Abstract])) OR ("bicycle"[Title/Abstract])** Sort by: **Most Recent**
15,97912:38:06#2

Search: **("bicycling"[Title/Abstract]) AND ("cycling"[Title/Abstract])** Sort by: **Most Recent**
27912:35:42#1

Search: **((((((((((("old people"[Title/Abstract]) OR ("older people"[Title/Abstract])) OR ("elderly"[Title/Abstract])) OR ("elders"[Title/Abstract])) OR ("aging"[Title/Abstract])) OR ("ageing"[Title/Abstract])) OR ("old men"[Title/Abstract])) OR ("old women"[Title/Abstract])) OR ("older persons"[Title/Abstract])) OR ("older adults"[Title/Abstract])) OR ("seniors"[Title/Abstract])** Sort by: **Most Recent**

"old people"[Title/Abstract] OR "older people"[Title/Abstract] OR "elderly"[Title/Abstract] OR "elders"[Title/Abstract] OR "aging"[Title/Abstract] OR "ageing"[Title/Abstract] OR "old men"[Title/Abstract] OR "old women"[Title/Abstract] OR "older persons"[Title/Abstract] OR "older adults"[Title/Abstract] OR "seniors"[Title/Abstract]
603,23412:32:10

Web of Science

☐ **ABSTRACT:**

12
#5 AND #10
21
Add to query
☐
11
#5 OR #10
350
Add to query
☐
10
#6 AND #9
337
Add to query
☐
9
#7 OR #8
12,513
Add to query
☐
8
AB=("bicycling" OR "biking" OR "bike" OR "bicycle")
12,513
Add to query
☐
7
AB=("bicycling" AND "cycling")
144
Add to query

☐

6

AB=("old people" OR "older people" OR "elderly" OR "elders" OR "aging" OR "ageing" OR "old men" OR "old women" OR "older persons" OR "older adults" OR "seniors")

450,596

Add to query

☐ **Title**

5

#1 AND #4 34

Add to query

☐

4

#2 OR #3

5,234

Add to query

☐

3

TI=("bicycling" OR "biking" OR "bike" OR "bicycle")

5,234

Add to query

☐

2

TI=("bicycling" AND "cycling")

6

Add to query

☐

1

TI=("old people" OR "older people" OR "elderly" OR "elders" OR "aging" OR "ageing" OR "old men" OR "old women" OR "older persons" OR "older adults" OR "seniors")

298,334

Add to query

Scopus

2,189 document results on 21/12/2021, 22:30

TITLE-ABS-KEY ((("bicycling" AND "cycling") OR ("bicycling" OR "biking" OR "bike" OR "bicycle")) AND ("old people" OR "older people" OR "elderly" OR "elders" OR "aging" OR "ageing" OR "old men" OR "old women" OR "older persons" OR "older adults" OR "seniors")

Cochrane library

Title, Abstract, Keyword

Date Run: 22/12/2021 05:57:42

Comment:

ID Search Hits

#1 ("old people" OR "older people" OR "elderly" OR "elders" OR "aging" OR "ageing" OR "old men" OR "old women" OR "older persons" OR "older adults" OR "seniors"): ti,ab,kw (Word variations have been searched) 768130

#2 ("bicycling" AND "cycling"): ti,ab,kw (Word variations have been searched) 2465

#3 ("bicycling" OR "biking" OR "bike" OR "bicycle"): ti,ab,kw (Word variations have been searched) 6155

#4 #2 OR #3 6155

#5 #1 AND #4 **3027** (3020 systamatic 7 trial)

SportDiscus

((("bicycling" AND "cycling") OR ("bicycling" OR "biking" OR "bike" OR "bicycle")) AND ("old people" OR "older people" OR "elderly" OR "elders" OR "aging" OR "ageing" OR "old men" OR "old women" OR "older persons" OR "older adults" OR "seniors")**No filter: 410**

PsychInfo

((("bicycling" AND "cycling") OR ("bicycling" OR "biking" OR "bike" OR "bicycle")) AND ("old people" OR "older people" OR "elderly" OR "elders" OR "aging" OR "ageing" OR "old men" OR "old women" OR "older persons" OR "older adults" OR "seniors")

No filter: 180

CINAHL

((("bicycling" AND "cycling") OR ("bicycling" OR "biking" OR "bike" OR "bicycle")) AND ("old people" OR "older people" OR "elderly" OR "elders" OR "aging" OR "ageing" OR "old men" OR "old women" OR "older persons" OR "older adults" OR "seniors")

No filter: 214 Duplicate:(33)

Total 181

Articles published from 23rd December 2021 to 18th June 2023 / "or" Dec 2021 to June 2023

List of 33 articles:PubMed :

A single bout of moderate-intensity aerobic exercise improves motor learning in premanifest and early Huntington's disease.
Andrews SC, Kämpf L, Curtin D, Hinder M, Wenderoth N, Stout JC, Coxon JP.Front Psychol. 2023 Mar 8;14:1089333. doi: 10.3389/fpsyg.2023.1089333. eCollection 2023

Enhancing Visual Exploration through Augmented Gaze: High Acceptance of Immersive Virtual Biking by Oldest Olds.
de'Sperati C, Dalmasso V, Moretti M, Høeg ER, Baud-Bovy G, Cozzi R, Ippolito J

Safer cycling in older age (SiFAr): effects of a multi-component cycle training. a randomized controlled trial.
Keppner V, Krumpoch S, Kob R, Rappl A, Sieber CC, Freiberger E, Siebentritt HM.BMC Geriatr. 2023 Mar 7;23(1):131. doi: 10.1186/s12877-023-03816-2

The Effects of Exergaming on Executive and Physical Functions in Older Adults With Dementia: Randomized Controlled Trial.
Wu S, Ji H, Won J, Jo EA, Kim YS, Park JJ.J Med Internet Res. 2023 Mar 7;25:e39993. doi: 10.2196/39993

PedaleoVR: Usability study of a virtual reality application for cycling exercise in patients with lower limb disorders and elderly people.
Rojo A, Castrillo A, López C, Perea L, Alnajjar F, Moreno JC, Raya R.PLoS One. 2023 Feb 22;18(2):e0280743. doi: 10.1371/journal.pone.0280743. eCollection 2023

Effects of exergame and bicycle exercise intervention on blood pressure and executive function in older adults with hypertension: A three-group randomized controlled study.
Hou HY, Chen J, Hai L, Wang P, Zhang JX, Li HJ

Web of Science

Effects of exergame and bicycle exercise intervention on blood pressure and executive function in older adults with hypertension: A three-group randomized controlled study

A Combined Intervention of Aerobic Exercise and Video Game in Older Adults: The Efficacy and Neural Basis on Improving Mnemonic Discrimination

Effects of acute aerobic exercise on mnemonic discrimination performance in older adults

PedaleoVR: Usability study of a virtual reality application for cycling exercise in patients with lower limb disorders and elderly people

SportDiscus

Association between cardiorespiratory fitness and cerebrovascular reactivity to a breath-hold stimulus in older adults: influence of aerobic exercise training.
Academic Journal
DuBose, Lyndsey E.; Weng, Timothy B.; Pierce, Gary L.; Wharff, Conner; Reist, Lauren; Hamilton, Chase; O'Deen, Abby; Dubishar, Kaitlyn; Lane-Cordova, Abbi; Voss, Michelle W., Journal of Applied Physiology Jun2022, Vol. 132 Issue 6, p1468 (English Abstract Available)

The effects of acute exercise on driving and executive functions in healthy older adults.
Academic Journal
Lebeau, Jean-Charles; Mason, Justin; Roque, Nelson; Tenenbaum, Gershon, International Journal of Sport & Exercise Psychology Jan2022, Vol. 20 Issue 1, p283 (English Abstract Available)

.
Acute Blood Pressure Effects in Older Adults with Hypertension After Different Modalities of Exercise: An Experimental Study.
Academic Journal
Cunha, Raphael M.; Arsa, Gisela; Oliveira-Silva, Iransé; Ferreira Rocha, Izabela; Machado Lehnen, Alexandre, Journal of Aging & Physical Activity Dec2021, Vol. 29 Issue 6, p952 (English Abstract Available)

Exercise Dose and Aerobic Fitness Response in Alzheimer's Dementia: Findings from the FIT-AD Trial.
Academic Journal
Salisbury, Dereck; Mathiason, Michelle A.; Yu, Fang, International Journal of Sports Medicine Sep2022, Vol. 43 Issue 10, p850

The ratio of heart rate to heart rate variability reflects sympathetic activity during incremental cycling exercise.
Academic Journal
Tanoue, Yukiya; Komatsu, Tomohiro; Nakashima, Shihoko; Matsuda, Takuro; Michishita, Ryoma; Higaki, Yasuki; Uehara, Yoshinari, European Journal of Sport Science Nov2022, Vol. 22 Issue 11, p1714 (English Abstract Available)

Moderate Intensity Cycling Combined with Cognitive Dual-task Improves Selective Attention.
Academic Journal
Kunzler, Marcos Roberto; Carpes, Felipe P., International Journal of Sports Medicine Jun2022, Vol. 43 Issue 6, p545 (English Abstract Available)

A Pilot Randomized Controlled Trial of Interval Training and Sleep Hygiene for Improving Sleep in Older Adults.
Academic Journal
Mahajan, Aashirwad; Mahajan, Satish; Tilekar, Swanand, Journal of Aging & Physical Activity Dec2021, Vol. 29 Issue 6, p993 (English Abstract Available)

CINAHL

Effect of Adding Motorized Cycle Ergometer Over Exercise Training on Balance in Older Adults with Dementia: A Randomized Controlled Trial.
Academic Journal
(includes abstract) Abbas, Rami L.; Saab, Ibtissam M.; Al-Sharif, Hassan K.; Naja, Nabil; El-Khatib, Ayman Experimental Aging Research, Mar/Apr2023; 49(2): 100-111. 12p. (Article - research, tables/charts, randomized controlled trial) ISSN: 0361-073X
.
Stationary Cycling Exercise Improved Manual Dexterity in Older Adults with Down Syndrome.
Academic Journal
(includes abstract) Chen, C.-C.; Ringenbach, S. D. R.; Arnold, N.; Nam, K. Journal of Developmental & Physical Disabilities, Aug2022; 34(4): 705-716. 12p. (Article - research, tables/charts) ISSN: 1056-263X

.
Acute Blood Pressure Effects in Older Adults with Hypertension After Different Modalities of Exercise: An Experimental Study.

Academic Journal

(includes abstract) Cunha, Raphael M.; Arsa, Gisela; Oliveira-Silva, Iransé; Ferreira Rocha, Izabela; Machado Lehnen, Alexandre Journal of Aging & Physical Activity, Dec2021; 29(6): 952-958. 7p. (Article - research, tables/charts, randomized controlled trial) ISSN: 1063-8652

Cochrane library

Feasibility and Effects of Virtual Reality Motor-Cognitive Training in Community-Dwelling Older People With Cognitive Frailty: Pilot Randomized Controlled Trial

RYC Kwan, JYW Liu, KNK Fong, J Qin, PK Leung, OSK Sin, PY Hon, LW Suen, MK Tse, CK Lai

Effect of Adding Motorized Cycle Ergometer Over Exercise Training on Balance in Older Adults with Dementia: a Randomized Controlled Trial

RL Abbas, IM Saab, HK Al-Sharif, N Naja, A El-Khatib

Effects of exergame and bicycle exercise intervention on blood pressure and executive function in older adults with hypertension: a three-group randomized controlled study

HY Hou, J Chen, L Hai, P Wang, JX Zhang, HJ Li

Cycle more with virtual reality: a proof of concept study in an institutionalised able-bodied geriatric population

G Loggia, A Gauthier, F Lemiere, J Drigny, A Desvergee, P Leconte, A Rue

The Effects of Exergaming on Executive and Physical Functions in Older Adults With Dementia: randomized Controlled Trial

S Wu, H Ji, J Won, EA Jo, YS Kim, JJ Park

Effects of exercise games on improving cognitive function and health promotion in the elderly with dementia

KCT0008238

https://trialsearch.who.int/Trial2.aspx?TrialID=KCT0008238, 2023 | added to CENTRAL: 30 April 2023 | 2023

Dual-task Cycling System on Cognitive Function for the Elderly

https://clinicaltrials.gov/show/NCT05384639, 2022 | added to CENTRAL: 30 June 2022 | 2022 Issue 06

Scopus

Study protocol for two pilot randomised controlled trials aimed at increasing physical activity using electrically assisted bicycles to enhance prostate or breast cancer survival

Safer cycling in older age (SiFAr): effects of a multi-component cycle training. a randomized controlled trial

A virtual reality-based endurance training program for COPD patients: acceptability and user experience

Effect of high intensity interval training on arterial stiffness in obese hypertensive women: a randomized controlled trial

The Effects of Exergaming on Executive and Physical Functions in Older Adults With Demer Randomized Controlled Trial

Virtual Reality Exercise Program Effects on Body Mass Index, Depression, Exercise Fun and Exercise Immersion in Overweight Middle-Aged Women: A Randomized Controlled Trial

PychInfo N/A
