# Supplementary material for: Acceptability and effectiveness of stationary bike intervention on health outcomes among older adults: a systematic review of intervention studies
Source: BMC Geriatr. 2026 Jan 13;26:130. doi: 10.1186/s12877-025-06757-0 (PMC12857070; doi:10.1186/s12877-025-06757-0)
Supplement: Supplementary file 4 — Supplementary Material 4. [file 12877_2025_6757_MOESM4_ESM.docx]

**Supplementary table 1.** Different health outcomes after stationary bike intervention

| **First Author**  **(Year of publication))** | **health outcomes after stationary bike intervention** | | | | | | | | | | | | | | | | |
| --- | --- | --- | --- | --- | --- | --- | --- | --- | --- | --- | --- | --- | --- | --- | --- | --- | --- |
|  | Executive function | | Cognition | | Memory | Aerobic capacity (VO_2_ max) | Cycling efficiency | | Quality Of Life (QOL) | Balance (BBS test) | Mobility (TUG  test) | Walking Capacity | Gait | Pain reduction | Lowering of BP | Lowering of HR |  |
|  | Colour trails test | Stroop a/c test | CR time (↑) (not improved) | CR time (↓) (improved) |  |  | With VA | Without VA |  |  |  |  |  |  |  |  |  |
| Abbas (2022) |  |  |  |  |  |  | +  *(p < 0.05*) | (-)  No change |  | + | (-) |  |  |  |  |  |  |
| [38] |  |  |  |  |  |  |  |  |  | *(p<0.05)* | *(p<0.05)* |  |  |  |  |  |  |
| Anderson- | + |  |  |  |  |  |  |  |  |  |  |  |  |  |  |  |  |
| Hanley, C. | *(p=0.002* |  |  |  |  |  |  |  |  |  |  |  |  |  |  |  |  |
| (2012) [39] | *)* |  |  |  |  |  |  |  |  |  |  |  |  |  |  |  |  |
| Anderson- | + |  |  |  |  |  |  |  |  |  |  |  |  |  |  |  |  |
| Hanley | *(p=0.007* |  |  |  |  |  |  |  |  |  |  |  |  |  |  |  |  |
| (2012) [40] | *)* |  |  |  |  |  |  |  |  |  |  |  |  |  |  |  |  |
| Anderson- |  | + |  |  |  |  |  |  |  |  |  |  |  |  |  |  |  |
| Hanley |  | *(p=0.049* |  |  |  |  |  |  |  |  |  |  |  |  |  |  |  |
| (2018) [42] |  | *)* |  |  |  |  |  |  |  |  |  |  |  |  |  |  |  |
| Anderson- |  |  |  |  |  |  | + | (-)  No change |  |  |  |  |  |  |  |  |  |
| Hanley |  |  |  |  |  |  | *(p=0.003* |  |  |  |  |  |  |  |  |  |  |
| (2011) [41] |  |  |  |  |  |  | *)* |  |  |  |  |  |  |  |  |  |  |
|  |  |  |  |  |  |  | *(↑Compet* |  |  |  |  |  |  |  |  |  |  |
|  |  |  |  |  |  |  | *itiveness* |  |  |  |  |  |  |  |  |  |  |
|  |  |  |  |  |  |  | *and* |  |  |  |  |  |  |  |  |  |  |
|  |  |  |  |  |  |  | *exercise* |  |  |  |  |  |  |  |  |  |  |
|  |  |  |  |  |  |  | *effort))* |  |  |  |  |  |  |  |  |  |  |
| Antunes |  |  |  |  | + | + |  |  |  |  |  |  |  |  |  |  |  |
| (2015) [18] |  |  |  |  | *(p <0.05)* | *(p <0.05)* |  |  |  |  |  |  |  |  |  |  |  |
| Barcelos | + | (-)  No change |  |  |  |  |  |  |  |  |  |  |  |  |  |  |  |
| (2015) [43] | *(p=0.02)* |  |  |  |  |  |  |  |  |  |  |  |  |  |  |  |  |

| Bellumori |  |  |  |  |  |  |  |  |  |  | + |  |  |  |  |  |
| --- | --- | --- | --- | --- | --- | --- | --- | --- | --- | --- | --- | --- | --- | --- | --- | --- |
| (2017) [44] |  |  |  |  |  |  |  |  |  |  | *(p <0.05)* |  |  |  |  |  |
| Briswalter |  |  |  |  |  |  |  | (-) *(p<0.05)* |  |  |  |  |  |  |  |  |
| (2014) [46] |  |  |  |  |  |  |  |  |  |  |  |  |  |  |  |  |
|  |  |  |  |  |  |  |  |  |  |  |  |  |  |  |  |  |
| Buccola |  |  |  |  |  | + |  |  |  |  |  |  |  |  | **+** |  |
| (1975) [45] |  |  |  |  |  | *(p <0.05)* |  |  |  |  |  |  |  |  | *(p <0.05)* |  |
| Callow(2022) [47] |  |  |  |  |  |  |  |  |  |  |  |  |  |  |  |  |
| Cicek (2020)  [48] |  |  |  |  |  |  |  |  |  | +  *(p=0.002)* | (-)  No change |  |  |  |  |  |
| Colombo |  |  |  |  |  |  |  |  |  |  |  | *+* |  |  |  |  |
| (2023) [49] |  |  |  |  |  |  |  |  |  |  |  | *(p<0.05)* |  |  |  |  |
| Cunha (2021  [19] |  |  |  |  |  |  |  |  |  |  |  |  |  |  | (-)  No change |  |
| D'Cunha  (2021) [33] |  |  |  |  |  |  | *(+)*  *(p=0.012*  *)*  *(↑ perceived exertion)* | (-)  No change |  |  |  |  |  |  |  |  |
| Emery |  |  |  |  |  | ++ |  |  |  |  |  |  |  |  |  |  |
| (1994) [20] |  |  |  |  |  | *(p <0.001)* |  |  |  |  |  |  |  |  |  |  |
| Ferrai (2004)  [21] |  |  |  |  |  | ++ |  |  | + |  |  |  |  |  |  |  |
|  |  |  |  |  |  | *(p=0.001****)*** |  |  |  |  |  |  |  |  |  |  |
|  |  |  |  |  |  |  |  |  | *(QOL* |  |  |  |  |  |  |  |
|  |  |  |  |  |  |  |  |  | *score:* |  |  |  |  |  |  |  |
|  |  |  |  |  |  |  |  |  | *7/9, p* |  |  |  |  |  |  |  |
|  |  |  |  |  |  |  |  |  | *value* |  |  |  |  |  |  |  |
|  |  |  |  |  |  |  |  |  | *did not* |  |  |  |  |  |  |  |
|  |  |  |  |  |  |  |  |  | *mention* |  |  |  |  |  |  |  |
|  |  |  |  |  |  |  |  |  | *)* |  |  |  |  |  |  |  |
| Ferraz (2018) [22] |  |  |  |  |  |  |  |  |  |  |  | *++ (p=0.001)* |  |  |  |  |
| Gitlin (1992)  [50] |  |  |  |  |  |  |  |  | *++ (p<0.00 1)* |  |  |  |  |  |  |  |
| Hill (2015) [51] |  |  |  |  |  |  |  |  |  |  |  |  | (-)  No change |  |  |  |
| Hou (2023) [23] |  |  |  |  | (+)  *(p=0.021*  ***)*** |  |  |  |  |  |  |  |  |  |  |  |
| Joyce (2014) [24] |  |  |  | *++* *(p<0.001)* |  |  |  |  |  |  |  |  |  |  |  |  |
| Katyal (2003) [25] |  |  |  |  |  | +  *(p <0.05)* |  |  |  |  |  |  |  |  |  |  |
| Kwan (2021)  [34] |  |  | +  *(p=0.01)*  *(Overall cognitive function)* | |  |  |  |  |  |  |  |  |  |  |  |  |
| Lebeau (2020) [26] |  | *++ (p=0.001*  *)* |  |  |  |  |  |  |  |  |  |  |  |  |  |  |
| Lee (2014) [10] |  |  |  |  |  |  |  |  |  | +  *(p<0.05)* |  |  |  |  |  |  |
| Loggia (2021) [52] |  |  |  |  |  |  | +  *(p <0.05)*  *(Repetitiv e participat ion)* |  |  |  |  |  |  |  |  |  |
| Lopez-Garcia (2019) [27] |  |  | ++  *(p<0.001)* |  |  |  | +  *(p <0.05)*  *(longer distance higher perceived effort)* | (-)  (lower effort, short distance) |  |  |  |  |  |  |  |  |
| Madden (2009) [28] |  |  |  |  |  | (-)  No change |  |  |  |  |  |  |  |  |  |  |
| Mahajan (2021) [53] |  |  |  |  |  |  |  | *+*  *(p <0.05* |  |  |  |  |  |  |  |  |
| Miki (2014) [54] |  |  | +  *(p=0.006)*  *(Overall cognitive function)* | |  |  |  |  |  |  |  |  |  |  |  |  |
| Morita (2013) [29] |  |  |  |  |  |  |  |  |  |  |  |  |  |  | +  *(p <0.05)*  *(Women)* |  |
| Nocera (2020) [30] |  |  |  |  |  | ++  *(p <0.001)* |  |  |  |  | +  *(p <0.03)* |  |  |  |  |  |
| Pauwels (2018) [55] |  |  |  |  |  |  |  |  |  |  |  |  |  | +  *(p=0.01)* |  |  |
| Posner (1992) [56] |  |  |  |  |  | ++  *(p <0.001)* |  |  |  |  |  |  |  |  |  |  |
| Rezasoltani (2020) [31] |  |  |  |  |  |  |  |  |  |  |  |  |  | ++  *(p*  *<0.001)* |  |  |
| Ridgel (2019) [57] |  |  |  |  |  |  | *+*  *(p =0.002)*  *(VA > non-VA)* | +  *(p = 0.012)* |  |  | +  *(p=0.002)* |  | +  *(p=0.012)* |  |  |  |
| Salisbury (2022) [58] |  |  |  |  |  | (+)  *(p=0.03****)*** |  |  |  |  |  |  |  |  |  |  |
| Schwarck (2021) [2] |  |  |  | +  *(Significan t but p*  *value was absent))* |  |  |  |  |  |  |  |  |  |  |  |  |
| Tollár (2019) [32] |  |  |  |  |  |  |  |  | (-)  No change | (-)  No change |  | (-)  No change |  |  |  |  |
| VanRoie (2017) [35] |  |  |  |  |  |  |  |  | ++ *(p<0.00 1)* |  |  |  |  |  |  |  |
| Willenheimer (1998) [60] |  |  |  |  |  | (-)  No change |  |  | +  *(p<0.01*  *)*  *(Men)* |  |  |  |  |  |  | (-)  *No change* |
| Wu (2023) [36] |  |  | +  *(p≤0.05)* |  |  |  |  |  |  |  |  |  |  |  |  |  |
| Yu (2011)  [37] |  |  |  |  |  |  |  |  |  |  |  |  |  |  |  | +  *(p=0.01)* |
| Yu (2021)  [61] | (-)  No change  (*Overall executive function)* | | (-)  No change  (*Overall cognitive function)* | | (-)  No change |  |  |  |  |  |  |  |  |  |  |  |

*N=* *45 studies tabulated out of 47 studies*

*Here p-value is considered as statistically significant (+) if, p is ≤ 0.05; highly significant (++) if, p is ≤0.001; and not significant if, p is > 0.05. Minus (-) signifies: Stationary bikes intervention failed to show any better improvement over other interventions among the study or trial group.*

*CR: Choice Reaction, VA: Virtual Aid, BP: Blood pressure, HR: Heart rate, BBS: Berg Balance Scale, TUG: Timed Up & Go, VO2 Max: Maximum oxygen capacity*

**Supplementary table 2.** Assessment of study quality of the 28 RCT studies according to PEDro scale

| **Study** | **Criterion 02:**  **Random allocation** | **Criterion 03:**  **Concealed allocation** | **Criterion 04:**  **Groups similar at baseline** | **Criterion 05:**  **Participant blinding** | **Criterion 06:**  **Therapist blinding** | **Criterion 07:**  **Assessor blinding** | **Criterion 08:**  **more than 85% of outcomes measured** | **Criterion 09:**  **Intention- to-treat analysis** | **Criterion 10:**  **Between- group difference reported** | **Criterion 11:**  **Point estimate and variability**  **reported** | **Total (0**  **to 10)** |
| --- | --- | --- | --- | --- | --- | --- | --- | --- | --- | --- | --- |
| Abbas et al. 2022 | Y | N | Y | N | Y | Y | Y | Y | Y | Y | 8 |
| Anderson- Hanley et al.  2012 | Y | N | N | Y | N | N | Y | Y | Y | Y | 6 |
| Anderson- Hanley et al. 2011 | Y | N | N | N | N | N | Y | Y | Y | Y | 5 |
| Anderson- Hanley et al. 2018 | Y | N | Y | N | N | N | Y | Y | Y | Y | 6 |
| Antunes et al. 2015 | Y | N | Y | N | N | Y | Y | Y | Y | Y | 7 |
| Barcelos et al. 2015 | Y | N | N | N | N | N | Y | Y | Y | Y | 4 |
| Bellumori et al. 2017 | Y | N | Y | N | N | N | Y | Y | Y | Y | 6 |
| D’Cunha et al. 2011 | Y | Y | Y | Y | N | Y | Y | Y | Y | Y | 9 |
| Ferraz et al. 2018 | Y | Y | Y | Y | Y | Y | Y | Y | Y | Y | 10 |
| Gitlin et al. 1992 | Y | N | Y | N | N | N | Y | Y | Y | Y | 6 |
| Hou et al. 2023 | Y | Y | N | Y | Y | Y | Y | Y | Y | Y | 9 |
| Kwan et al. 2021 | Y | N | N | N | Y | Y | Y | Y | Y | Y | 7 |

| **Study** | **Criterion 02:**  **Random allocation** | **Criterion 03:**  **Concealed allocation** | **Criterion 04:**  **Groups similar at baseline** | **Criterion 05:**  **Participant blinding** | **Criterion 06:**  **Therapist blinding** | **Criterion 07:**  **Assessor blinding** | **Criterion 08:**  **more than 85% of outcomes measured** | **Criterion 09:**  **Intention- to-treat analysis** | **Criterion 10:**  **Between- group difference reported** | **Criterion 11:**  **Point estimate and variability**  **reported** | **Total (0**  **to 10)** |
| --- | --- | --- | --- | --- | --- | --- | --- | --- | --- | --- | --- |
| Lebeau et al. 2020 | Y | N | N | N | N | N | Y | Y | Y | Y | 5 |
| Lo´ pez- Garci´a et al. 2019 | Y | N | N | N | N | N | Y | Y | Y | Y | 5 |
| Madden et al. 2009 | Y | Y | Y | Y | Y | Y | Y | Y | Y | Y | 10 |
| Mahajan et al. 2021 | Y | Y | Y | Y | Y | Y | Y | Y | Y | Y | 10 |
| Miki et al. 2014 | Y | Y | Y | Y | Y | Y | Y | Y | Y | Y | 10 |
| Morita et al. 2013 | Y | N | N | N | N | N | Y | Y | Y | Y | 5 |
| Nocera et al. 2020 | Y | Y | Y | Y | N | N | Y | Y | Y | Y | 8 |
| Posner et al. 1992 | Y | N | N | N | N | N | Y | Y | Y | Y | 5 |
| Rezasoltani et al. 2020 | Y | Y | Y | Y | Y | Y | Y | Y | Y | Y | 10 |
| Ridgel et al. 2019 | Y | N | Y | N | N | N | Y | Y | Y | Y | 6 |
| Salisbury ey al. 2022 | Y | Y | N | Y | Y | N | Y | Y | Y | Y | 8 |
| Tollar et al. 2019 | Y | Y | N | Y | N | Y | Y | Y | Y | Y | 8 |
| Van Roie et al. 2017 | Y | Y | N | Y | N | Y | Y | Y | Y | Y | 8 |
| Willenheimer et al. 1998 | Y | N | N | N | N | N | Y | Y | Y | Y | 5 |

| **Study** | **Criterion 02:**  **Random allocation** | **Criterion 03:**  **Concealed allocation** | **Criterion 04:**  **Groups similar at baseline** | **Criterion 05:**  **Participant blinding** | **Criterion 06:**  **Therapist blinding** | **Criterion 07:**  **Assessor blinding** | **Criterion 08:**  **more than 85% of outcomes measured** | **Criterion 09:**  **Intention- to-treat analysis** | **Criterion 10:**  **Between- group difference reported** | **Criterion 11:**  **Point estimate and variability**  **reported** | **Total (0**  **to 10)** |
| --- | --- | --- | --- | --- | --- | --- | --- | --- | --- | --- | --- |
| Wu et al. 2023 | Y | Y | Y | Y | Y | Y | Y | Y | Y | Y | 10 |
| Yu et al. 2021 | Y | Y | N | N | N | Y | Y | Y | Y | Y | 7 |

**Supplementary table 3.** Risk of Bias assessment of 28 RCT studies according to the

Cochrane risk of bias tool, version 2

| Criterion | Randomization process | Deviations from intended interventions | Missing outcome data | Measurement of the outcome | Selection of the reported result | Overall |
| --- | --- | --- | --- | --- | --- | --- |
|  |  |  |  |  |  |  |
| Abbas et al. 2022 | 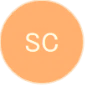 | 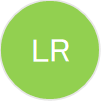 | 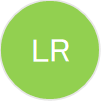 | 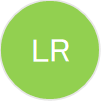 | 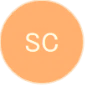 | 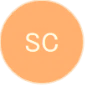 |
| Anderson-Hanley et al. 2012 | 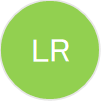 | 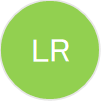 | 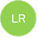 | 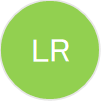 | 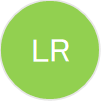 | 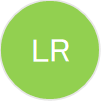 |
| Anderson-Hanley et al. 2011 | 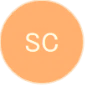 | 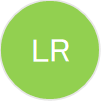 | 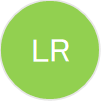 | 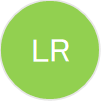 | 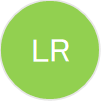 | 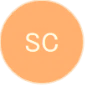 |
| Anderson-Hanley et al. 2018 | 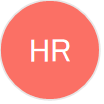 | 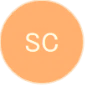 | 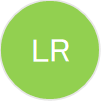 | 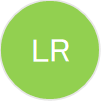 | 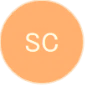 | 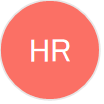 |
| Antunes et al. 2015 | 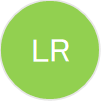 | 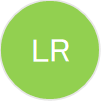 | 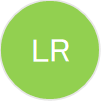 | 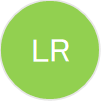 | 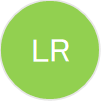 | 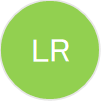 |
| Barcelos et al. 2015 | 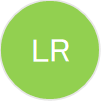 | 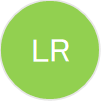 | 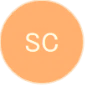 | 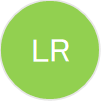 | 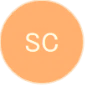 | 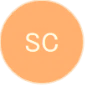 |
| Bellumori et al. 2017 | 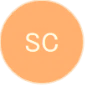 | 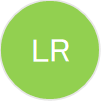 | 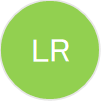 | 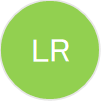 | 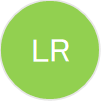 | 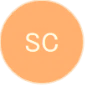 |
| D’Cunha et al. 2011 | 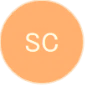 | 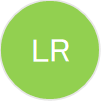 | 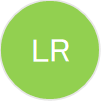 | 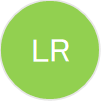 | 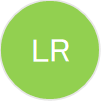 | 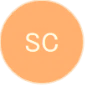 |
| Ferraz et al. 2018 | 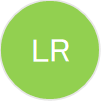 | 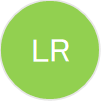 | 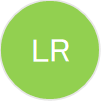 | 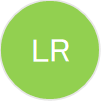 | 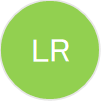 | 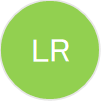 |
| Gitlin et al. 1992 | 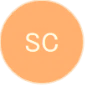 | 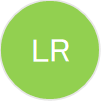 | 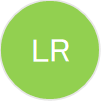 | 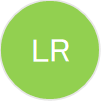 | 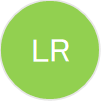 | 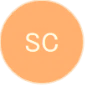 |
| Hou et al. 2023 | 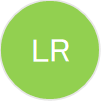 | 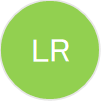 | 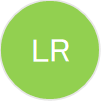 | 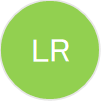 | 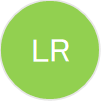 | 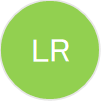 |
| Kwan et al. 2021 | 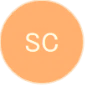 | 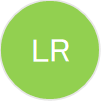 | 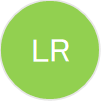 | 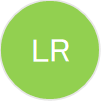 | 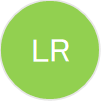 | 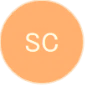 |
| Lebeau et al. 2020 | 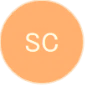 | 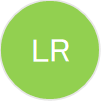 | 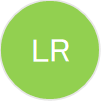 | 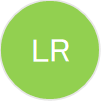 | 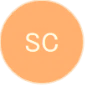 | 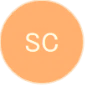 |
| Lo´ pez-Garcı´a et al. 2019 | 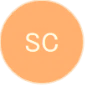 | 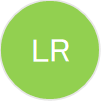 | 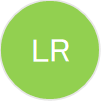 | 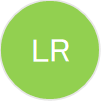 | 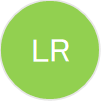 | 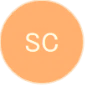 |
| Madden et al. 2009 | 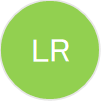 | 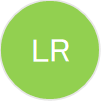 | 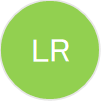 | 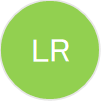 | 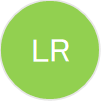 | 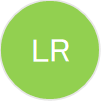 |
| Mahajan et al. 2021 | 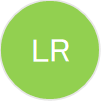 | 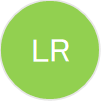 | 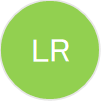 | 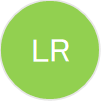 | 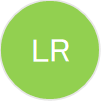 | 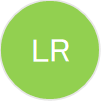 |
| Miki et al. 2014 | 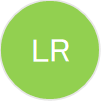 | 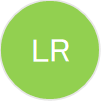 | 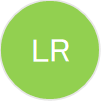 | 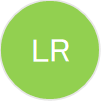 |  |  |
| Morita et al. 2013 |  |  |  |  |  |  |
| Nocera et al. 2020 |  |  |  |  |  |  |
| Posner et al. 1992 |  |  |  |  |  |  |
| Rezasoltani et al. 2020 |  |  |  |  |  |  |
| Ridgel et al. 2019 |  |  |  |  |  |  |
| Salisbury et al. 2022 |  |  |  |  |  |  |
| Tollar et al. 2019 |  |  |  |  |  |  |
| Roie et al. 2017 |  |  |  |  |  |  |
| Willenheimer et al. 1998 |  |  |  |  |  |  |
| Wu et al. 2023 |  |  |  |  |  |  |
| Yu et al. 2023 |  |  |  |  |  |  |

**Supplementary table 4.** Quality assessment of the 19 non-randomized controlled trials according to Newcastle-Ottawa Scale

| **Study (year)** | **Selection** | | | | **Comparability** | **Outcome** | | | **Score** |
| --- | --- | --- | --- | --- | --- | --- | --- | --- | --- |
|  | Representativeness of the exposed cohort | Selection of the non- exposed cohort | Ascertainment of exposure | Outcome of interest was not present at start of  study | Comparability of cohorts on the basis of the design or  analysis | Assessment of outcome | Adequate follow-up duration | Adequate follow-up rate |  |
| **1. Anderson (2012)** | ★ | ★ | - | ★ | ★ | ★ | ★ | ★ | 7 |
| **2. Brisswalter (2014)** | ★ | ★ | ★ | - | ★ | ★ | ★ | ★ | 7 |
| **3. Buccola (1975)** | - | - | - | ★ | ★ | ★ | ★ | ★ | 5 |
| **4. Callow (2022)** | ★ | ★ | ★ | ★ | ★★ | ★ | ★ | ★ | 9 |
| **5. Cicek (2020)** | ★ | ★ | ★ | ★ | ★★ | ★ | ★ | ★ | 9 |
| **6. Colombo (2023)** | ★ | ★ | ★ | ★ | ★★ | ★ | ★ | ★ | 9 |
| **7. Cunha (2021)** | ★ | ★ | ★ | ★ | ★★ | ★ | ★ | ★ | 9 |
| **8. Emery (1994)** | ★ | ★ | ★ | ★ | ★ | ★ | ★ | ★ | 8 |
| **9. Ferrai (2004)** | ★ | ★ | - | ★ | ★★ | ★ | ★ | ★ | 8 |
| **10. Gaesser (2018)** | ★ | ★ | ★ | - | ★ | ★ | ★ | ★ | 7 |
| **11. Hill (2015)** | ★ | ★ | ★ | ★ | ★★ | ★ | ★ | ★ | 9 |
| **12. Joyce (2014)** | ★ | ★ | ★ | ★ | ★★ | ★ | ★ | ★ | 9 |
| **13. Katyal (2003)** | ★ | ★ | ★ | ★ | ★★ | ★ | ★ | ★ | 9 |
| **14. Lee (2014)** | ★ | ★ | ★ | ★ | ★ | ★ | ★ | ★ | 8 |
| **15. Loggia (2021)** | ★ | ★ | ★ | ★ | ★ | ★ | ★ | ★ | 8 |
| **16. Pauwels (2018)** | - | ★ | ★ | ★ | ★★ | ★ | - | ★ | 7 |
| **17. Schwarck (2021)** | ★ | ★ | ★ | ★ | ★★ | ★ | ★ | ★ | 9 |
| **18. Yu (2011)** | ★ | ★ | ★ | ★ | ★★ | ★ | ★ | ★ | 9 |
| **19. Yu (2013)** | ★ | **-** | ★ | **-** | **-** | **-** | **-** | ★ | 3 |

**Supplementary Figure 1.** Trend of publication

*QOL=Quality of life, VR=Virtual reality*

9

8

7

6

5

4

3

2

1

0

1975-1980 1981-1986 1987-1992 1993-1998 1999-2004 2005-2010 2011-2016 2017-2023

Cognitive function

Motor and Balance

Physiological and psychological changes

Cardiovascular system

Cycling efficiency and QOL Executive function

Musculoskeletal VR induced training Feasibility and effectiveness

**Appendix:1** **TIDieR framework of VA-enhanced SB intervention among older adults (≥ 60 years) for 9 included RCTs studies**

| **TIDieR Item** | López-García (2019) [27] | D’Cunha (2021) [33] | Abbas **(2022) [38]** | Anderson-Hanley (2012) [40] | Anderson-Hanley **(2011)**  **[41]** | Anderson-Hanley **(2018)**  **[42]** | Barcelos **(2015)**  **[43]** | Miki (2014)  **[54]** | Ridgel **(2019)**  **[57]** |
| --- | --- | --- | --- | --- | --- | --- | --- | --- | --- |
| **1. Brief name** | Active videogame cycling | Virtual group cycling | Motorized stationary cycling with/without feedback | Cybercycle exergame | Cybercycle (VA) vs traditional stationary cycling | Cybercycle with cognitive challenge | ACE exergame cycling | Speed-feedback cycling therapy | VA-enhanced cycling |
| **2. Why (Rationale)** | Improve cognitive flexibility and reaction time while exercising | Increase engagement and social connectedness in people with dementia | Stimulate engagement in dementia Comparator: Motorized cycle without VA | Combine aerobic and cognitive stimulation to improve physical and neuropsychological outcomes in older adults with diabetes | Enhance cognition and physical effort. Comparator: Traditional stationary bike (no VA) | Add cognitive challenge to aerobic cycling. Comparator: Traditional exercise bike (no VA) | Combine cognitive and aerobic training. Comparator: Traditional stationary cycling | Maintain cognitive function during cancer therapy | Increase motor drive via immersive cycling. Comparator: Standard stationary cycling |
| **3. What (Materials)** | Stationary bike + active videogame software | Stationary bikes connected via virtual screen simulating group rides | Motorized cycle device with digital feedback | Stationary bike with built-in virtual environment (cybercycle) | Cybercycle vs standard stationary bike | Cybercycle with “exer-tour” or “exer-score” mode | Stationary bike with exergame software | Bicycle ergometer with real-time speed feedback monitor | Stationary bike with immersive VA headset |
| **4. What (Procedures)** | Complete active videogame cycling sessions, self-paced | Group cycling sessions virtually linked, conversational prompts | Structured pedaling with/without feedback | Ride set distances in an interactive virtual environment | Routine cycling; VA group used cybercycle | Same cycling dose, cognitive tasks in VA mode | Cycling plus simultaneous cognitive tasks | Cycle at prescribed speeds with visual speed feedback | VA tasks while pedaling at set cadence |
| **5. Who provided** | Exercise lab staff | Allied health staff and researchers | Trained therapists in care facility | Research staff (exercise lab) | University exercise lab staff | Research assistants | Research staff | Oncology rehabilitation therapists | Research therapists/technicians |
| **6. How (Delivery)** | Individually supervised | Small group, virtually connected | Individually supervised sessions | Individually supervised | Individually in lab, with HR monitoring | Individually, supervised | Individually, supervised | Individually supervised | Individually, supervised |
| **7. Where** | University setting | Residential aged care facility | Long-term care facility | University laboratory | University/community fitness sites | University setting | University lab | Hospital oncology ward | Clinical research lab |
| **8. When & how much** | Acute: 2 sessions × 60 min; also 25 min bout; 3-month intervention at 60% HRmax | 6 weeks, 2–3/wk, 30–40 min | 3sessions /wk × 30 min for 6 weeks | 3 months, 2–3/wk, ~45 min | 2-3 sessions /wk × 45 min for 3 months | 3 sessions /wk × 30-45 min for 3 months | 2-3 sessions /wk × 20-30 min for 12 weeks | 4 weeks, 2–3/wk, 30 min | 2-3 sessions /wk × 30-40 min for 8 weeks |
| **9. Tailoring** | Self-paced at set target speeds (~1.8 m/s) | Intensity adjusted to tolerance and cognitive level | Adjusted to participant comfort | Self-paced at 60–70% HRmax | Self-paced, optional HR targets (60–70% HRmax) | Tasks scaled for cognitive challenge | Progressive cognitive challenge levels | Speed targets tailored to baseline capacity | Cadence (60-80 rpm) adjusted to tolerance |
| **10. Modifications** | None reported | None reported | None reported | None reported | None reported | None reported | None reported | None reported | None reported |
| **11. Planned fidelity** | Software distance logs | Staff session logs, observation | Session logs, supervision | Attendance logs, HR monitoring | Supervised + device logs | Supervised sessions | Supervised sessions | Session logs | Supervised + cadence logs |
| **12. Actual fidelity** | Full adherence during sessions | Moderate adherence (~80%) | High adherence (>80%) | >85% adherence | >85% adherence | 50% | 42% | High adherence | High adherence (percentage unreported) |

ACE=aerobic and cognitive exercise, VA=virtual aid, HR=heart rate, RPM= revolutions per minute, WK=week, TIDieR=Template for Intervention Description and Replication.

**Appendix: 2 PRISMA 2020 checklist (all 27 reporting items) for Systematic Reviews and Meta-Analyses**

| **Section and Topic** | **Item #** | **Checklist item** | **Location where item is reported** |
| --- | --- | --- | --- |
| **TITLE** | | |  |
| Title | 1 | Identify the report as a systematic review. | Page: 1, Lines: 1 - 3 |
| **ABSTRACT** | | |  |
| Abstract | 2 | See the PRISMA 2020 for Abstracts checklist. | Page: 1-3, Lines: 23 -6 |
| **INTRODUCTION** | | |  |
| Rationale | 3 | Describe the rationale for the review in the context of existing knowledge. | Page: 4-5, Lines: 15-10 |
| Objectives | 4 | Provide an explicit statement of the objective(s) or question(s) the review addresses. | Page: 5, Lines: 10-18 |
| **METHODS** | | |  |
| Eligibility criteria | 5 | Specify the inclusion and exclusion criteria for the review and how studies were grouped for the syntheses. | Page:6-7, Lines: 3 -9 |
| Information sources | 6 | Specify all databases, registers, websites, organisations, reference lists and other sources searched or consulted to identify studies. Specify the date when each source was last searched or consulted. | Page:7, Lines: 11 -24 |
| Search strategy | 7 | Present the full search strategies for all databases, registers and websites, including any filters and limits used. | Supplementary file S1 |
| Selection process | 8 | Specify the methods used to decide whether a study met the inclusion criteria of the review, including how many reviewers screened each record and each report retrieved, whether they worked independently, and if applicable, details of automation tools used in the process. | Page:7-8, Lines: 25 - 10 |
| Data collection process | 9 | Specify the methods used to collect data from reports, including how many reviewers collected data from each report, whether they worked independently, any processes for obtaining or confirming data from study investigators, and if applicable, details of automation tools used in the process. | Page:8, Lines: 11 -23 |
| Data items | 10a | List and define all outcomes for which data were sought. Specify whether all results that were compatible with each outcome domain in each study were sought (e.g. for all measures, time points, analyses), and if not, the methods used to decide which results to collect. | Page:8-9, Lines: 24- 4 |
|  | 10b | List and define all other variables for which data were sought (e.g. participant and intervention characteristics, funding sources). Describe any assumptions made about any missing or unclear information. | Page:8-9, Lines: 24- 4 |
| Study risk of bias assessment | 11 | Specify the methods used to assess risk of bias in the included studies, including details of the tool(s) used, how many reviewers assessed each study and whether they worked independently, and if applicable, details of automation tools used in the process. | Page:9, Lines: 5- 21 |
| Effect measures | 12 | Specify for each outcome the effect measure(s) (e.g. risk ratio, mean difference) used in the synthesis or presentation of results. | Page:9-10, Lines: 22-7 |
| Synthesis methods | 13a | Describe the processes used to decide which studies were eligible for each synthesis (e.g. tabulating the study intervention characteristics and comparing against the planned groups for each synthesis (item #5)). | Page:10, Lines: 8-12 |
|  | 13b | Describe any methods required to prepare the data for presentation or synthesis, such as handling of missing summary statistics, or data conversions. | Page:10, Lines: 8-12 |
|  | 13c | Describe any methods used to tabulate or visually display results of individual studies and syntheses. | Page:10, Lines: 8-12 |
|  | 13d | Describe any methods used to synthesize results and provide a rationale for the choice(s). If meta-analysis was performed, describe the model(s), method(s) to identify the presence and extent of statistical heterogeneity, and software package(s) used. | Page:10, Lines: 8-12 |
|  | 13e | Describe any methods used to explore possible causes of heterogeneity among study results (e.g. subgroup analysis, meta-regression). | Page:10, Lines: 8-12 |
|  | 13f | Describe any sensitivity analyses conducted to assess robustness of the synthesized results. | Page:10, Lines: 8-12 |
| Reporting bias assessment | 14 | Describe any methods used to assess risk of bias due to missing results in a synthesis (arising from reporting biases). | Page:10, Lines:13-14 |
| Certainty assessment | 15 | Describe any methods used to assess certainty (or confidence) in the body of evidence for an outcome. | Page:10, Lines:15-16 |
| **RESULTS** | | |  |
| Study selection | 16a | Describe the results of the search and selection process, from the number of records identified in the search to the number of studies included in the review, ideally using a flow diagram. | Page:10, Lines 19-23; Page: 45 |
|  | 16b | Cite studies that might appear to meet the inclusion criteria, but which were excluded, and explain why they were excluded. | Page:10, Lines 19-23; Page: 45 |
| Study characteristics | 17 | Cite each included study and present its characteristics. | Page:10-11, Lines: 24-19 |
| Risk of bias in studies | 18 | Present assessments of risk of bias for each included study. | Page:11-21, Lines 20-21 |
| Results of individual studies | 19 | For all outcomes, present, for each study: (a) summary statistics for each group (where appropriate) and (b) an effect estimate and its precision (e.g. confidence/credible interval), ideally using structured tables or plots. | Page:12-17, Lines 22-16 |
| Results of syntheses | 20a | For each synthesis, briefly summarise the characteristics and risk of bias among contributing studies. | Page:17-18, Lines 17-11 |
|  | 20b | Present results of all statistical syntheses conducted. If meta-analysis was done, present for each the summary estimate and its precision (e.g. confidence/credible interval) and measures of statistical heterogeneity. If comparing groups, describe the direction of the effect. | Page:17-18, Lines 17-11 |
|  | 20c | Present results of all investigations of possible causes of heterogeneity among study results. | Page:17-18, Lines 17-11 |
|  | 20d | Present results of all sensitivity analyses conducted to assess the robustness of the synthesized results. | Page:17-18, Lines 17-11 |
| Reporting biases | 21 | Present assessments of risk of bias due to missing results (arising from reporting biases) for each synthesis assessed. | Page:18, Lines 14-17 |
| Certainty of evidence | 22 | Present assessments of certainty (or confidence) in the body of evidence for each outcome assessed. | Page 18, Lines: 12-19 |
| **DISCUSSION** | | |  |
| Discussion | 23a | Provide a general interpretation of the results in the context of other evidence. | Page 18-21, Lines 22-09 |
|  | 23b | Discuss any limitations of the evidence included in the review. | Page: 22, Lines 10-21 |
|  | 23c | Discuss any limitations of the review processes used. | Page: 22, Lines 10-21 |
|  | 23d | Discuss implications of the results for practice, policy, and future research. | Page 20- 21, Lines 23-9; Page: 22-23: Lines 24-14 |
| **OTHER INFORMATION** | | |  |
| Registration and protocol | 24a | Provide registration information for the review, including register name and registration number, or state that the review was not registered. | Page:5-6, Lines: 25-01 |
|  | 24b | Indicate where the review protocol can be accessed, or state that a protocol was not prepared. | Page:5-6, Lines: 25-01 |
|  | 24c | Describe and explain any amendments to information provided at registration or in the protocol. | No changes needed |
| Support | 25 | Describe sources of financial or non-financial support for the review, and the role of the funders or sponsors in the review. | Page: 24, Lines:3-7 |
| Competing interests | 26 | Declare any competing interests of review authors. | Page: 24, Lines:1-2 |
| Availability of data, code and other materials | 27 | Report which of the following are publicly available and where they can be found: template data collection forms; data extracted from included studies; data used for all analyses; analytic code; any other materials used in the review. | Page 23, Lines 21-25 |

*PRISMA: Preferred Reporting Items for Systematic Reviews and Meta-Analyses. From:*  Page MJ, McKenzie JE, Bossuyt PM, Boutron I, Hoffmann TC, Mulrow CD, et al. The PRISMA 2020 statement: an updated guideline for reporting systematic reviews. BMJ 2021;372:n71. doi: 10.1136/bmj.n71. This work is licensed under CC BY 4.0. To view a copy of this license, visit <https://creativecommons.org/licenses/by/4.0/>
